# Ulcerative colitis: signaling pathways, therapeutic targets and interventional strategies

**DOI:** 10.1038/s41392-025-02345-1

**Published:** 2026-02-11

**Authors:** Jinhao Jia, Ying Liu, Dan Wang, Zhaohai Pan, Qiusheng Zheng, Jun Lu, Chao Liang, Defang Li

**Affiliations:** 1 Featured Laboratory for Biosynthesis and Target Discovery of Active Components of Traditional Chinese Medicine, School of Traditional Chinese Medicine, Binzhou Medical University, Yantai, China; 2 State Key Laboratory of Southwestern Chinese Medicine Resources, School of Pharmacy, Chengdu University of Traditional Chinese Medicine, Chengdu, China; 3 Department of Systems Biology, School of Life Sciences, Southern University of Science and Technology, Shenzhen, China; 4 Institute of Integrated Bioinfomedicine and Translational Science (IBTS), School of Chinese Medicine, Hong Kong Baptist University, Hong Kong, China; 5 College of Pharmacy, Hebei University of Chinese Medicine, Shijiazhuang, China

**Keywords:** Gastrointestinal diseases, Molecular medicine

## Abstract

Ulcerative colitis (UC) is the most common chronic inflammatory disease of the intestinal tract in clinical practice, and long-term chronic inflammation leads to repeated damage to and repair of the colonic mucosa, which may progress to malignancy through atypical hyperplasia. However, there are currently no fully targeted drugs for the treatment of UC. In this review, we discuss several cellular processes, such as autophagy, endoplasmic reticulum stress, mitochondrial dysfunction, macrophage polarization, ferroptosis and the Th/Treg cell balance, which are associated with the occurrence and development of UC. Many molecular targets and signaling pathways, such as nuclear factor kappa-B (NF-κB), phosphatidylinositol 3 kinase/protein kinase B (PI3K/AKT), Wnt/β-catenin, adenosine 5’-monophosphate-activated protein kinase (AMPK), toll-like receptor (TLR), Janus kinase/signal transducer and activator of transcription (JAK/STAT), long noncoding RNAs (lncRNAs), and microRNAs (miRNAs), play crucial roles in the progression of UC. We also summarize the common treatment strategies for UC, including lifestyle interventions, aminosalicylic acid preparations, corticosteroid drugs, biologics, fecal microbiota transplantation, and other drugs for symptomatic treatment. This review provides a detailed theoretical basis for the pathology and treatment of UC. Future research could focus on optimizing the treatment plan and achieving more precise and personalized treatment with multiple targets in multiple aspects.

## Introduction

Ulcerative colitis (UC) is a chronic immune inflammatory disease that occurs in the gastrointestinal tract. The World Health Organization describes it as one of the most difficult-to-treat diseases in modern times and one of the most common causes of colorectal cancer.^1–4^ The number of people with UC is estimated to be ~224 cases per 100,000 people per year globally, with an annual growth rate of 2%.^5^ The annual incidence ranges from 8.8 to 23.1 per 100,000 person-years in North America, 0.6–24.3 per 100,000 person-years in Europe, and 7.3–17.4 per 100,000 person-years in Oceania, with a prevalence in adults in their 30 s and 40 s.^5–7^ However, there is no consensus on the cause of this disease. Most medical scientists attribute UC to a combination of immunomodulatory factors, genetic factors and environmental factors that cause ulcerative lesions in the intestinal mucosa.^8–10^ Long-term dietary irregularities and metabolic dysfunction caused by obesity have also been demonstrated to increase the prevalence of UC.^11,12^ Consequently, the clinical treatment of UC has been extremely challenging. Given that UC recurrence causes pain, as well as great financial and psychological pressures,^13,14^ it is an increasing burden on public health worldwide.^15^

With ongoing advances in medical research, the treatment options for UC are continually improving and expanding. Although meaningful results have been achieved in understanding the pathogenesis and therapeutic targets of UC, progress in the treatment of UC remains slow. No drugs have been specifically developed for the clinical treatment of UC, and UC is still considered a clinically incurable disease.^16^ Consequently, more specific therapeutic targets and more effective therapeutic drugs are urgently needed. In this review, to provide a more solid theoretical basis for UC treatment, we summarize the current targeted therapy drug options and the signaling pathways associated with UC pathogenesis.

## Clinical manifestations, diagnosis and epidemiological studies of ulcerative colitis

UC, first described in 1859, is usually confined to the colon, where persistent superficial inflammation of the upper mucosa of the colon develops, leading to colonic ulceration and bleeding.^17^ In the late 19th century, the English physician Samuel Wilks and the English surgeon Thomas Henry Allchin first used the term “chronic ulcerative colitis” to describe the disease, which they systematically observed and described clinically.^18^ UC is characterized by ulceration of the intestinal mucosal layer in the rectum and colon, which in turn affects the entire colon, resulting in dysregulation of intestinal immune factors and disruption of the structure and function of the intestinal barrier.^19–21^ With the continuous advancement of medical technology, the understanding of UC has gradually deepened. UC patients are divided into three subtypes according to their molecular and cellular characteristics: patients with obvious enrichment of epithelial activation-related pathways (subtype A; epithelial proliferative UC), patients with mixed UC (subtype B), and patients with significant immune cell and proinflammatory characteristics (subtype C; immune-activated UC). In subtype B patients, the pathways of subtypes A and C are both activated.^22^

Patients in subtype A have active proliferation of epithelial cells, significant enrichment of associated signaling pathways such as the Wnt/β-catenin pathway, and relatively strong repair and regeneration of epithelial cells. Patients often present with mild to moderate symptoms, and epithelial cell proliferation is observed via endoscopy, which may be accompanied by colonic swelling.^23^ Patients with subtype B exhibit a mixture of features, including both epithelial proliferation and some degree of immune activation. Patients may present diverse symptoms and endoscopic manifestations, along with an inflammatory response of varying severity.^24^ Patients with subtype C disease have significant immune cell infiltration and proinflammatory features, especially a significant increase in the activity of immune cells such as lymphocytes and macrophages. Patients usually present with severe symptoms, with significant inflammation and ulceration visible on endoscopy.^25^ Although each subtype has unique characteristics, they may overlap in some respects. For example, some patients with subtype A disease may progressively change to subtype C, especially if the disease progresses or is affected by specific triggers.^26^

UC is a chronic inflammatory disease that usually manifests itself clinically as a slowly progressive process and can be broadly divided into four stages.^27^ In the initial stages, local inflammation and ulceration of the intestinal mucosa occur, and the most common symptoms include abdominal pain, diarrhea, blood in the stool, anemia, and fatigue.^28,29^ Symptoms may initially be mild, but they gradually worsen as the disease progresses.^30^ During recurrent episodes of inflammation, symptoms may persist for weeks or months. During the second stage, patients may experience intermittent symptom relief and exacerbation, accompanied by abnormal mucin expression and infiltration of inflammatory cells.^31^ Over time, a third phase of stabilization, erosion and ulceration of the colonic mucosal epithelium may be accompanied by bleeding and the secretion of large amounts of mucus, with relatively stable symptoms that require long-term treatment and management to prevent recurrence.^32^ At this stage, some patients may experience pathological changes, such as intestinal stricture and perforation, as well as serious complications, including severe abdominal pain, severe diarrhea, and high fever.^33^ In the fourth stage (colorectal cancer), the colon wall is thickened, intestinal narrowing occurs, and the patient may experience persistent abdominal pain, blood in the stool, and changes in bowel behavior, thus seriously affecting the patient’s life and health.^34^

Early diagnosis is essential for the timely treatment and management of this disease. However, in the initial stages, the patient’s symptoms are not obvious, which can delay patient evaluation and diagnosis.^35^ With the development of endoscopic technology, doctors can evaluate the condition of the patient’s colonic mucosa more clearly, which enables more accurate diagnosis and treatment of UC.^36^ At present, colonoscopy is one of the most commonly used methods to confirm UC via direct observation of the pathology of the colonic mucosa or collection of samples for tissue biopsy.^37^ However, the invasive nature of endoscopy often causes physical pain as well as psychological stress to patients.^38^

With the development of science and technology, AI-assisted methods can also be applied for the early diagnosis of UC, resulting in increased diagnostic accuracy.^39,40^ Similarly, ultrasonic testing can be used to detect UCs.^41^ Noninvasive serological tests, such as tests for eosinophils,^42^ calprotectin,^43^ and C-reactive protein,^44,45^ are helpful in the diagnosis of UC. Furthermore, substantial evidence indicates that there are significant abnormalities in the levels of mucin,^46^ serum adiponectin,^47^ lipids,^48^ and erythrocyte ferritin^49^ in patients with UC. Gut microbiota testing and fecal immunochemical testing have also been used in the diagnosis of UC.^50,51^

In recent years, many new promising UC biomarkers have been discovered for the early diagnosis of UC.^52–54^ Amino acid metabolites and short-chain fatty acid metabolites have been identified as specific biomarkers for UC, and leucine-rich α2-glycoprotein (LRG) has been developed as a serum biomarker of disease progression in UC patients.^55,56^ The overexpression of matrix metalloproteinase genes has been identified in the colon tissue of UC patients and colon cancer patients.^57^ Other novel biomarkers include noncoding RNAs,^58^ miRNAs,^59,60^ and lncRNAs^61^ (e.g., miR-21, miR-146a, miR-155, lncRNA HOTAIR, and lncRNA NEAT1). The discovery of these biomarkers may facilitate a more timely and accurate diagnosis of UC, which is essential for the health and quality of life of patients.

Patients with UC for an extended period often suffer from malnutrition or anemia due to insufficient dietary intake.^62–64^ During long-term disease treatment, patients may also experience anxiety and depression.^65,66^ Inflammation and long-term ulceration of the colonic mucosa caused by UC may lead to abnormal proliferation and differentiation of colonic mucosal cells, thereby increasing the risk of colorectal cancer.^67^ Evidence suggests that up to 10% of UC patients with colitis who have not been cured for more than 10 years may develop cancer.^68^ The World Health Organization predicts that by 2030, there will be more than 2.2 million new cases of colorectal cancer and more than 1.1 million cumulative deaths, and the trend is increasing each year.^69^ Increasing research has shown that in addition to the increased incidence of colorectal cancer, UC patients have an increased risk of extraintestinal cancers,^70^ such as pancreatic cancer,^71^ prostate cancer,^72^ hepatocarcinoma,^73^ and anal cancer.^74^ Moreover, UC patients also have an increased prevalence of chronic diseases, such as chronic kidney disease,^75^ atherosclerosis,^76^ and mental illness.^77,78^ Surprisingly, previous studies have confirmed the protective effect of smoking on UC, but it has likewise been suggested that active smoking can increase the incidence of adverse effects in the treatment of UC patients.^79,80^

Therefore, UC is an undeniable disease that seriously affects people’s life and health. To better treat this disease, the following three aspects should be taken into consideration. First, to achieve early diagnosis and treatment, the publicity of the factors and hazards of UC should be strengthened to make everyone aware of its serious consequences. Second, to achieve precise clinical diagnosis of UC, more reasonable diagnostic criteria, such as noninvasive serological tests, gut microbiota testing, and detection of specific metabolites, should be developed and applied in clinical practice. Third, research on the cellular processes and key therapeutic targets should focus on finding new therapeutic targets and developing more effective targeted drugs for UC.

## Cellular processes associated with ulcerative colitis development

UC is characterized by alterations in multiple cellular processes that underlie the pathology of the disease. In this section, we review evidence of the involvement of autophagy, endoplasmic reticulum stress, mitochondrial dysfunction, and macrophage polarization in UC pathology. We also outline current studies that support the involvement of the gut microbiota and mesenchymal cells in the development and progression of UC, with implications for therapeutic development.

### Autophagy

**Fig. 1:**
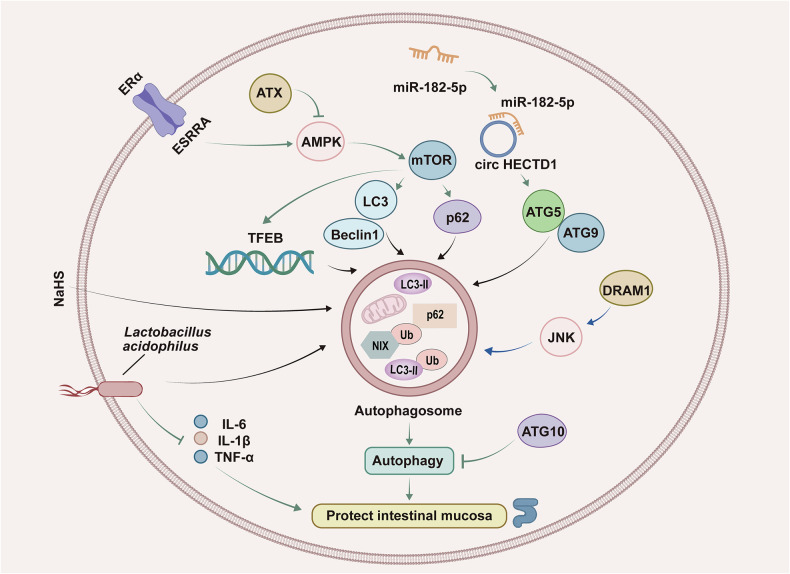
Regulation of autophagy in UC. Autophagy is a cellular process of self-degradation and recycling of intracellular components that promotes cell renewal and repair. The formation of autophagosomes is an important hallmark of autophagy. CircHECTD1 overexpression promotes the formation of autophagosomes and reduces colonic injury by inhibiting miR-182-5p and activating AMPK/mTOR signaling. The regulation of both estrogen-related receptor alpha (ERα) and autochemotactic proteins can promote autophagy via the AMPK pathway, which in turn maintains the integrity of the mucosal barrier. The probiotic *Lactobacillus acidophilus* can also promote the formation of autophagosomes. Furthermore, DRAM1 inhibits the formation of autophagosomes and attenuates the symptoms of UC by positively regulating JNK. ESRRA estrogen-related receptor alpha, ATX autotaxin, LC3 microtubule-associated protein light chain 3, ATG5 autophagy-related gene 5, ATG9 autophagy-related gene 9, TFEB transcription factor EB, JNK c-Jun N-terminal kinase

Autophagy is a regulatory process by which damaged proteins and organelles are removed from cells to promote cell renewal and repair.^81^ Studies have shown that autophagy, characterized by the expression of autophagy markers (ATG5, ATG7, LC3, etc.), may play a crucial role in the pathogenesis and inflammatory process of UC, helping to reduce the inflammatory response and promote the repair of the intestinal mucosa.^82^ However, there is currently insufficient clinical evidence to suggest that autophagy can directly treat UC. The molecular mechanisms by which autophagy is confirmed to be involved in the occurrence and development of UC are shown in Fig. 1.

Estrogen-associated receptor α promotes intestinal homeostasis by activating autophagy through the AMPK/mTOR signaling axis to protect colon tissue from inflammatory responses and mitochondrial dysfunction.^83^ Autotaxin inhibits autophagy via the AMPK pathway, thereby disrupting the mucosal barrier and aggravating intestinal inflammation, and AMPK agonists can significantly ameliorate this process, preventing intestinal inflammation and maintaining the integrity of the mucosal barrier.^84^ The overexpression of circHECTD1 leads to increased expression levels of p62, ATG5, and ATG9 via the inhibition of miR-182-5p, which activates AMPK/mTOR signaling and reduces colonic injury and inflammation by promoting autophagy.^85^ DNA damage-regulated autophagy modulator 1 (DRAM1) is essential for inducing autophagy and apoptosis and is highly expressed in UC patients.^86^ DRAM1 participates in the regulation of autophagy and apoptosis in intestinal epithelial cells by positively regulating JNK, and knockdown of DRAM1 can lead to a decrease in the phosphorylation levels of c-Jun and JNK, inhibit the formation of autophagosomes, and alleviate the symptoms of UC.^87^ In the UC rat model, NaHS can promote autophagy by regulating the levels of autophagy-related proteins, including increasing the LC3-II/LC3-I ratio and decreasing the level of p62; inhibiting the expression of IL-6, IL-1β and TNF-α; and decreasing the expression of the neutrophil infiltration marker MPO, which exacerbates intestinal injury.^88^ Moreover, the probiotic *Lactobacillus acidophilus* can maintain the homeostasis of inflammatory factors and improve intestinal barrier dysfunction in vitro and in vivo by promoting autophagy.^89^ ATG10 is an additional protein associated with autophagy that is highly expressed in UC patients.^90^ Taken together, these findings indicate that autophagy is involved in the occurrence and development of UC and that the inhibition of autophagy could be a therapeutic strategy for treating UC by alleviating intestinal inflammation.

### Endoplasmic reticulum stress

The endoplasmic reticulum is an important organ within the cell that is involved in regulating the synthesis, folding, and modification of intracellular proteins.^91^ In UC patients, endoplasmic reticulum function may be impaired, leading to abnormal accumulation of intracellular proteins and exacerbation of the cellular inflammatory response.^92^ Endoplasmic reticulum stress treatment mainly improves the symptoms of UC by regulating endoplasmic reticulum function and reducing the abnormal accumulation of intracellular proteins, thereby inhibiting the inflammatory response of cells.^93^ However, ER stress therapy is still in the exploratory stage and has not yet been widely used. There are three classical signal transduction pathways involved in the development of endoplasmic reticulum stress: IRE1, PERK, and ATF6.^94^ In the colonic mucosa of UC patients, one or more of the three ER stress pathways are activated, resulting in unbalanced protein synthesis and abnormal protein folding, which in turn exacerbates the inflammatory response and intestinal barrier disruption of UC.^95,96^

Excessive endoplasmic reticulum stress leads to an increase in trypsin activity, which in turn stimulates PAR2 and PAR4 receptors, increases intestinal permeability, destroys the function of the intestinal barrier, and promotes the development of inflammatory processes.^97^ An imbalance in phospholipid metabolism in the intestine can also induce the endoplasmic reticulum stress response and promote the apoptosis of intestinal epithelial cells in UC mice, resulting in abundant inflammatory cell infiltration and increased intestinal permeability.^98^ In addition, Muc2 gene mutation in mice can induce endoplasmic reticulum stress in intestinal epithelial cells, and the misfolding of intrinsic proteins in the intestinal epithelium triggers the inflammatory response of intestinal epithelial cells, thereby destroying the structure and function of the intestinal barrier in mice and promoting the progression of UC by affecting the intestinal microbiota.^99^ Conversely, IL-20 can reduce endoplasmic reticulum stress in intestinal epithelial cells by activating ERK1/2, thus improving the pathological state of UC mice.^100^ Overexpression of the multifunctional adiponectin omentin-1 inhibits ER stress in the colon tissue of UC mice and attenuates colon injury and inflammatory cell infiltration.^101^ Therefore, the inhibition of ER stress is also a therapeutic strategy for treating UC by alleviating intestinal inflammation.

### Mitochondrial dysfunction

Mitochondria are organelles that are involved mainly in the energy metabolism process of the cell, especially the tricarboxylic acid cycle, which produces the energy required by the cell.^102^ Studies have shown that mitochondrial dysfunction can lead to the occurrence of intestinal inflammation,^103,104^ and excessive mitochondrial division inhibits the repair of the intestinal mucosa by disrupting butyrate metabolism in intestinal epithelial cells.^105^ HSF2 can regulate the mitochondrial phagocytosis of intestinal epithelial cells via the PARL/PINK1/Parkin pathway, removing damaged mitochondria, decreasing the levels of mtROS in cells, suppressing intestinal mucosal inflammation, and maintaining the intestinal environment balance in UC.^106^ Oxidative stress is caused by skewing of the redox balance in the intracellular and extracellular environments.^107^ Studies have shown that improving intestinal mucosal barrier function and regulating inflammatory signaling pathways by inhibiting oxidative stress may help reduce the inflammatory response in UC patients and disrupt the intestinal barrier.^108,109^

### Macrophage polarization

**Fig. 2:**
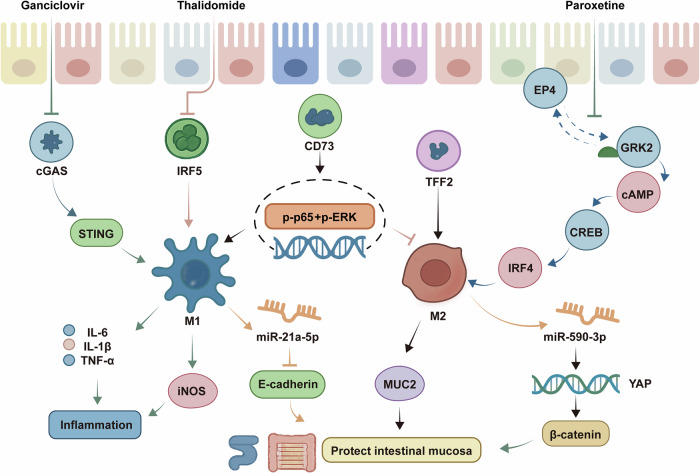
Macrophage polarization for targeted therapy of UC. Macrophage polarization is an important regulatory process in the immune response, and M1-type and M2-type macrophages play key roles in inflammatory and repair processes. M1-type macrophages promote the expression of inflammatory factors during immune regulation and aggravate the inflammatory progression of UC. M1 macrophage exocytosis decreases E-cadherin in the intestinal barrier through the secretion of miR-21a-5p, which disrupts the intestinal barrier structure and function. M2-type macrophages increase the levels of anti-inflammatory factors and promote the expression of mucins and tight junction proteins during immunomodulation, which in turn protects the structure and function of the intestinal barrier. miR-590-3p, which is secreted by M2 macrophage-derived exosomes, is translocated from macrophages to the intestinal epithelium and activates the YAP/β-catenin pathway, thereby promoting intestinal epithelial cell proliferation and UC mucosal healing. cGAS Cyclic guanosine monophosphate-adenosine monophosphate synthase, STING stimulator of interferon genes, IRF5 Interferon Regulatory Factor 5, CD73 Cluster of differentiation 73, CREB cAMP-response element binding protein, ‌YAP Yes-associated protein, iNOS inducible nitric oxide synthase

Macrophages are immune cells that play important roles in the body’s immune response.^110^ Macrophage polarization refers to the process by which macrophages are stimulated by different signals in various microenvironments to change their phenotype and function.^111^ The regulation of the polarization state of macrophages affects their role in the inflammatory process, which regulates the immune response of the intestinal mucosa in UC.^112–114^ The molecular mechanisms by which macrophage polarization is involved in the development of UC are shown in Fig. 2.

Macrophages can be differentiated into two main types on the basis of the signals received: classically activated M1 macrophages and alternatively activated M2 macrophages.^115^ M1 macrophages are involved in inflammatory and immune responses, releasing inflammatory cytokines that are involved in killing microorganisms and modulating immune responses.^116^ In contrast, M2 macrophages are associated with tissue repair and immune regulation, and they maintain tissue homeostasis by inhibiting inflammatory responses.^117,118^ Promoting M1 macrophage polarization and inhibiting M2 polarization promotes the intestinal inflammatory response, as it significantly increases the expression of CD73 in the colonic mucosal tissues of UC patients. Furthermore, M1 macrophage-derived exosomes reduce E-cadherin expression in the intestinal barrier by secreting miR-21a-5p, which exacerbates intestinal inflammation in UC model mice and destroys the structure and function of the intestinal barrier. In contrast, inhibiting the expression of miR-21a-5p can significantly restore intestinal epithelial cell damage in mice.^119^ The miR-590-3p secreted by M2 macrophage exosomes is transferred from macrophages to intestinal epithelial cells, activating the YAP/β-catenin pathway and thereby promoting the proliferation of intestinal epithelial cells and wound healing and alleviating intestinal mucosal injury.^120^ IRF4 also regulates macrophage M2 polarization by increasing IL-10 expression, promotes colonic mucosal epithelial cell proliferation, inhibits inflammation, and promotes tissue repair.^121^

The promotion of M2 macrophage polarization and the inhibition of M1 macrophage polarization have gradually emerged as recognized new methods for the treatment of UC.^122–124^ Restoring the balance between M1 and M2 macrophages not only regulates the expression of inflammatory factors but also maintains the physiological function of the intestinal barrier, regulating the dynamic balance of the intestinal microbiota to ameliorate UC.^125–127^ The antiviral drug ganciclovir has been shown to improve the intestinal inflammatory response and intestinal mucosal injury in UC mice by inhibiting STING activity in macrophages.^128^ Additionally, thalidomide inhibits M1 macrophage polarization by targeting the transcription factor IRF5, reduces the release of inflammatory signals, significantly reduces the secretion of the proinflammatory cytokines IL-12 and IFN-γ, and promotes intestinal mucosal healing.^129^ Paroxetine has also been shown to inhibit the inflammatory response in UC by blocking the interaction between G protein-coupled receptor kinase 2 (GRK2) and prostaglandin E2 receptor 4 (EP4), thereby reducing cyclic adenosine monophosphate (cAMP) levels and regulating M2 macrophage polarization.^130^

### Mesenchymal stem cells

Mesenchymal stem cells (MSCs) are adult stem cells with potential multidirectional differentiation ability. They were originally discovered in bone marrow but were later found in many other adult tissues, such as adipose tissue and placenta. MSCs also regulate the immune response, reduce inflammation, and promote tissue repair, which increase their interest in the treatment of immune-related diseases such as UC.^131–133^ MSCs have been shown to directly degrade Keap1 and activate Nrf2 activity, which increases the levels of HO-1 and NQO-1, inhibits the infiltration of colonic inflammatory cells and the expression of proinflammatory cytokines, and maintains the permeability of the mucosal barrier of colon epithelial cells.^134^

Bone marrow MSCs are the most common type of MSC. They have been shown to inhibit the expression of IFN-γ, IL-6, IL-1β, and IL-2; increase the expression of IL-4; and reduce colon inflammation and intestinal epithelial apoptosis by inhibiting the levels of Bax and caspase 9 in colon tissue.^135^ Further studies have suggested that hypoxic conditions increase the production of HIF-1α in bone marrow MSCs, which significantly improves the ability of bone marrow MSCs to inhibit DNA damage and apoptosis.^136^ In a UC rat model, pretreatment of bone marrow MSCs with IL-6 increased their ability to inhibit inflammatory factors.^137^ In addition, adipose MSCs, hair follicle MSCs, and dental pulp MSCs reduce inflammatory responses, promote mucosal healing, and improve intestinal barrier function.^138^ Among them, adipose MSCs play a role in the treatment of UC by inhibiting M1 macrophage polarization.^139^ Therefore, the use of MSCs could gradually become a good alternative for the treatment of UC.

### The gut microbiota

The gut microbiota refers to the microbial community that exists in the human intestine. It includes fungi, bacteria, viruses, and other microorganisms. These microorganisms form a large and complex ecosystem in the human gut and play important regulatory roles in human health and disease.^140^ In recent years, research on gut microbes has become more extensive, and people have gradually realized the close relationship between gut microbes and human health.^141,142^ Owing to the close interaction between the gut microbiota and the immune system, modulating the gut microbiota may be an effective way to treat and manage UC.^143–145^

The relative abundance of the gut microbiota is dynamic.^146,147^ UC patients have been shown to have significantly diminished gut microbiota diversity, with an increase in the abundance of harmful bacteria and a decrease in the abundance of beneficial bacteria, which affects the immune regulation and inflammatory response of the gut, thereby exacerbating symptoms and promoting the progression of the disease.^148^ A healthy F/B ratio is essential for maintaining intestinal homeostasis.^149^ Reducing the number of harmful *Firmicutes* bacteria while increasing the number of *Bacteroidetes* helps to restore intestinal homeostasis and improve intestinal permeability.^150–153^ In animal models with reduced F/B ratios, the expression of mucin and ZO-1 in the intestinal barrier, as well as the expression of anti-inflammatory factors, is increased, whereas the expression of the proinflammatory cytokines TNF-α, IL-1β, and IL-6 is inhibited, and disease symptoms are largely reversed.^154–156^ Furthermore, in the process of intestinal microbial reorganization, as the F/B ratio changes, the infiltration of inflammatory cells gradually decreases, and the structure and function of the intestinal barrier are gradually restored.^157,158^

*Lactobacillus*, a group of beneficial gram-positive lactic acid bacteria, is abundant in the intestine and contributes to improving the intestinal inflammatory response.^159–162^ Studies have shown that *Lactobacillus* can upregulate tight junction proteins, protect the integrity of the epithelial barrier, and enhance the function of the intestinal barrier by regulating the expression of aryl hydrocarbon receptors and NRF2.^163^ *Lactobacillus* can also increase the expression of HO-1 by activating NRF2, thereby suppressing the transcription of NF-κB and effectively improving intestinal inflammation and oxidative stress.^164^ In addition, *Lactobacillus* can promote the expression of ZO-1 and occludin, inhibit the activation of macrophages and the secretion of IL-17 by CD4 + T cells in colon tissue, and improve the overall gut microbiota while exerting anti-inflammatory effects.^165,166^ *B. lactis* BL-99 can regulate the composition of the intestinal microbiota, increase species richness, and reduce intestinal inflammation by regulating the intestinal microecological environment and inhibiting the expression levels of IL-1β and IL-6.^167^ *Lactobacillus* L15 and *Lactobacillus* C4 can also protect the integrity of the intestinal barrier by decreasing the expression levels of proinflammatory cytokines and upregulating the expression of tight junction proteins and mucin-2 (MUC2).^168,169^ As a nutrient, butyrate can reduce the secretion of inflammatory cytokines and stimulate the growth of probiotics (such as *Bifidobacterium lactis*). This effectively inhibits colonic inflammation and related histological changes.^170^ Notably, 5-aminosalicylic acid has been shown to have a significant regulatory effect on *Lactobacillus*, which may explain the pharmacological benefit of 5-aminosalicylic acid in UC treatment.^171^

*Akkermansia* is a bacterium commonly found in the intestines of humans and other animals that survives by breaking down and utilizing components in the intestinal mucus, with a crucial probiotic role in regulating gut health and immune function.^172,173^ Studies have shown that the addition of *Akkermansia* may help reduce the symptoms of UC, reduce intestinal inflammation and improve intestinal mucosal barrier function.^174–176^ In the UC animal model, the more severe the colon injury was, the lower the abundance of *Akkermansia*. Supplementation with *Akkermansia* helps increase the expression of mucin, promotes the proliferation and differentiation of goblet cells in the intestinal epithelium, and effectively protects the structure and function of the intestinal barrier.^177–179^ Aspartic acid (Asp) is an essential energy source for the intestinal mucosa, and studies have shown that Asp can increase the abundance of *Akkermansia* in the intestine by regulating RIPK1 signaling, maintaining the integrity of the intestinal barrier, and promoting colonic mucosal functional repair.^180^ Supplementation with vitamin A also increases the abundance of *Akkermansia* in the intestine of UC mice, promoting the expression of mucin and tight junction proteins in colon tissue and facilitating the recovery of the intestinal barrier.^181^ Changes in *Akkermansia* abundance have been demonstrated to improve the structure of the gut microbiota and alleviate colonic inflammation by affecting intestinal purine metabolism.^182^

*Escherichia coli* can cause gastrointestinal infections and other local tissue/organ infections under certain conditions.^183,184^ Studies have shown that the abnormal growth of *E. coli* can destroy the structure and function of the intestinal barrier and promote the occurrence and development of UC.^185–187^ When mast cells in the gastrointestinal tract are deficient in antitumor-associated G protein-coupled receptor X2 (MRGPRX2), *E. coli* in the intestinal tract proliferate, resulting in intestinal inflammatory cell infiltration and intestinal barrier damage.^188^ Moreover, *Escherichia coli* LF82 infection aggravates intestinal inflammation and intestinal mucosal barrier damage in UC by affecting the intestinal microbiota composition and indirectly regulating the balance of Th17 and Treg cell differentiation.^189^ Therefore, inhibition of the abundance of *E. coli* helps to regulate intestinal homeostasis and enhance the integrity of the intestinal barrier, thereby reducing colitis symptoms.^190,191^

*Fusobacterium nucleatum* is a common gram-negative nonsporous anaerobic bacillus. It is a harmful parasite in the oral cavity, upper gastrointestinal tract and intestines of humans and animals.^192^ Studies have shown that *Fusobacterium nucleatum* promotes the occurrence and development of UC by destroying the normal intestinal structure, downregulating the level of tight junction proteins, promoting the apoptosis of intestinal epithelial cells, increasing the expression of inflammatory cytokines, and disrupting the balance of the intestinal microbiota.^193^ In addition, *Fusobacterium nucleatum* can activate the RIPK1-mediated apoptosis pathway in UC, resulting in the destruction of tight junctions between cells, which in turn promotes the destruction of the intestinal barrier, inhibits the renewal of intestinal epithelial cells, and accelerates the progression of UC.^194^ Moreover, many studies have confirmed that *Shigella* is a common harmful pathogenic bacterium that leads to chronic inflammation of the colon and promotes the disease progression of UC.^195–197^

UC patients usually have an imbalance in the abundance of beneficial bacteria and harmful bacteria in the intestinal microbiota, which affects the intestinal microenvironment and results in damage to intestinal epithelial cells and the intestinal barrier.^198–200^ Given the importance of the gut microbiota in UC, researchers have explored ways to restore balance. A recent study revealed that it is possible to restore the microbial balance in the gut by transplanting stool samples from healthy donors into the recipient’s intestine.^201,202^ In an animal model of UC, fecal microbiota transplantation (FMT) significantly improved the α diversity of the intestinal microbiota, changed the composition of the intestinal microbiota, and played a role in UC treatment by inhibiting inflammatory factors and protecting the intestinal barrier.^203–206^ Studies have shown that FMT in combination with probiotic therapy at a ratio of 9:1 can significantly reduce intestinal barrier damage, decrease the levels of TNF-α and IL-6 in colon tissue, and alleviate UC symptoms.^207^ FMT can also reduce the release of IL-6 by regulating the NF-κB pathway and thus alleviate the intestinal inflammatory response in UC mice.^208^ Additionally, FMT can regulate the homeostasis of cytokines, significantly reducing susceptibility to colitis in germ-free mice.^209^

Taken together, these findings indicate that the gut microbiota can be used not only as a reliable diagnostic criterion but also as a biological agent for UC treatment. Some beneficial bacteria, such as Lactobacillus, Bifidobacteria, and *Bacillus licheniformis*, have been used as medications to regulate the intestinal environment.

## Signaling pathways driving ulcerative colitis development and their associated therapeutic targets

Given the complexity of UC, a comprehensive understanding of the signaling pathways that regulate UC pathogenesis is needed for the identification of potential therapeutic targets and the development of new treatments. Below, we describe several of the many signaling pathways involved in UC, including the PI3K/Akt, NF-κB, Wnt/β-catenin, AMPK, TLR, and STAT pathways, each of which is associated with potential therapeutic targets. We also describe lncRNAs and miRNAs as epigenetic regulators with potential diagnostic and targeting ability.

### NF-κB signaling pathway

**Fig. 3:**
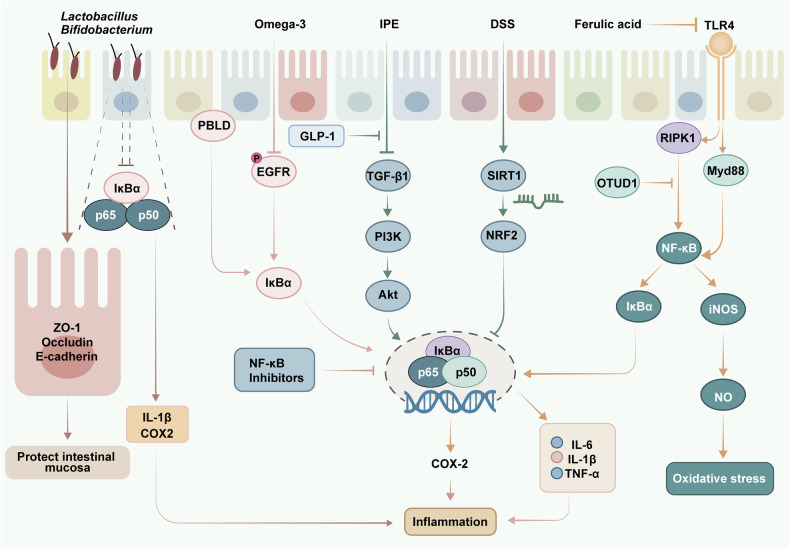
Key targets and upstream/downstream mediators of the NF-κB pathway in UC. The NF-κB pathway is a common inflammatory factor signaling pathway. External stimulation leads to the activation of the IκB kinase complex, resulting in the phosphorylation and ubiquitination degradation of the IκB protein, the activation of NF-κB-related proteins (p50 and p65), and ultimately the activation of NF-κB. Activation of the NF-κB pathway promotes the expression of inflammatory factors (IL-6, IL-1β, TNF-α, and COX2) and induces inflammatory responses. In addition, NF-κB signaling can affect the structure and function of the intestinal mucosa by regulating the expression of tight junction proteins. It also regulates oxidative stress by modulating the iNOS/NO pathway. Moreover, NF-κB inhibitors can alter disease progression in UC by directly inhibiting NF-κB-related proteins or inhibiting signaling molecules upstream of the NF-κB signaling pathway, such as TLRs and PI3K/Akt. IPE intestinal permeability enhancer, GLP-1 glucagon-like peptide-1, ZO-1 zonula occludens-1, OTUD1 OTU domain-containing protein 1, RIPK1 receptor-interacting protein kinase 1, COX-2 cyclooxygenase-2

The NF-κB signaling pathway plays a pivotal role in biological processes such as the immune response, cell proliferation, and apoptosis.^210,211^ The activation of NF-κB signaling is usually initiated by external stimuli such as cytokines or bacterial lipopolysaccharides, which bind to their corresponding receptors and trigger downstream signaling pathways. Receptor activation leads to activation of the IκB kinase complex, which results in phosphorylation and ubiquitination degradation of the IκB protein, ultimately activating NF-κB.^212^ There is increasing evidence that inhibition of the NF-κB signaling pathway can alleviate the symptoms of UC and thus play a beneficial therapeutic role.^213,214^ The key targets of the NF-κB signaling pathway and their related regulatory mechanisms are shown in Fig. 3.

In an animal study, inhibition of the NF-κB pathway led to a significant reduction in the expression of serum proinflammatory cytokines, including IL-6, IL-1β and TNF-α, as well as an increase in the expression of the tight junction proteins occludin and ZO-1, thereby improving the function and structure of the intestinal mucosa.^215^ High expression of NO is a characteristic of UC, and inhibition of the NF-κB pathway can also inhibit the expression of IL-6 and iNOS, thereby reducing the content of NO and protecting the intestinal mucosa.^216^ In dextran sulfate sodium (DSS)-induced colitis mice, a lack of phenazine biosynthesis-like domain-containing protein (PBLD) in the colon epithelium led to excessive activation of NF-κB. This was accompanied by increased secretion of chemokines and cytokines and increased immune cell infiltration in the colon. In contrast, PBLD activators significantly inhibited the activation of the NF-κB pathway.^217^ In another study, the deubiquitinating enzyme OTUD1 inhibited the NF-κB pathway by interacting with RIPK1 and inhibited the excessive production of the proinflammatory cytokines TNF-α, IL-6, and IL-1β in UC, thereby inhibiting colon inflammation.^218^

NF-κB inhibitors can inhibit NF-κB through different mechanisms, such as blocking the nuclear translocation of NF-κB, inhibiting the activation of NF-κB, and antagonizing the NF-κB signaling pathway.^219,220^ Anthocyanins reduce the phosphorylation of NF-κB p65 and downregulate the expression of NF-κB target genes and proteins, such as COX-2 and iNOS, which improves DSS-induced colitis.^221^ Furthermore, two natural substances, nerodol and citrus peel polyphenols, have been shown to improve the pathological state of UC mice and protect the integrity of the intestinal barrier by suppressing the activation of the NF-κB pathway.^222,223^ Eicosapentaenoic acid, an omega-3 fatty acid found in fish oil, inhibits the expression of IL-6 and IFN-γ by inhibiting the phosphorylation of TGF-β1 and EGFR, thereby inhibiting the nuclear translocation of NF-κB.^224^ Additionally, eicosapentaenoic acid ethyl ester enhances the antioxidant capacity of UC rats by promoting the expression of the SIRT1 protein, thereby activating the Nrf2 pathway and inhibiting the expression of NF-κB to regulate downstream inflammatory cytokines.^225^ A similar effect was found for vasoactive peptides, which inhibit the expression of NF-κB by activating the Nrf2 pathway and exerting anti-inflammatory effects. Moreover, FA improves the anti-inflammatory and antioxidant capacity of UC, which is also closely related to its ability to suppress the TLR4/NF-κB and NF-κB/iNOS/NO pathways.^226^

Aberrant sialylation can promote CD4^+^ T-cell activation, leading to the occurrence of UC, while deficiency of the α-2,6-sialyltransferase ST6GAL1 inhibits the process of sialylation and limits CD4^+^T cell activation by suppressing the expression of NF-κB, subsequently decreasing the production of proinflammatory cytokines and alleviating UC.^227^ The probiotics *Bifidobacterium* BGN4 and *Lactobacillus fermentum* HFY06 can also mediate the differentiation of T cells, inhibit the expression of IL-1β and COX2, promote the expression of the anti-inflammatory cytokine IL-10, enhance epithelial barrier function and reduce damage to the intestinal barrier by inhibiting the NF-κB signaling pathway.^228–230^ Dietary neuroside ganglioside inhibits NF-κB activation by regulating phospholipase transport and regulates inflammatory signaling and the expression of tight junction proteins, thus improving the integrity of the intestinal barrier.^231^ Similarly, exogenous supplementation with inhibitor of differentiation-2 (ID2) increases the level of Muc2 mRNA and the expression of Claudin-1 and ZO-1 by inhibiting the activation of neutrophil NF-kB; inhibits the inflammatory cytokines IL-1β, IL-6 and TNF-α; and reduces the infiltration of macrophages and neutrophils in the colon, thus restoring the integrity of the intestinal barrier.^232^ The long-chain monounsaturated fatty acid nervonic acid has also been demonstrated to significantly inhibit the phosphorylation of the IκB protein, thereby inhibiting the expression of COX-2, iNOS, IL-1β and TNF-α in UC mice.^233^

Sodium-glucose cotransporter-2 (SGLT2) inhibitors are commonly used for type 2 diabetes treatment, and in recent years, SGLT2 inhibitors canagliflozin has been shown to suppress IL-6 and IL-1β levels and MPO enzyme activity in the colon of UC rats by downregulating the expression of NF-κB p65, thereby reducing colonic inflammatory cell infiltration and oxidative stress.^234^ The nonsteroidal anti-inflammatory drug diclofenac can significantly reduce the protein expression level of NF-κB p65 and the phosphorylation level of IκB-α, effectively protecting the structure and function of the intestinal barrier by suppressing the iNOS/NF-κB pathway and activating the expression of HO-1.^235^ Ambroxol hydrochloride inhibits IL-6 and TNF-α by inhibiting the NF-κB signaling pathway while increasing the expression of IL-10 and reducing the infiltration of neutrophils and the apoptosis of intestinal epithelial cells.^236^ Moreover, the mucosal regulator carbocisteine has a similar effect.^237^ The therapeutic effects of the amino salicylic acid class drug sulfalazine,^238^ the antibiotics azithromycin and buspirone,^239^ the opioid agonist human opiorphin,^240^ the PDE5 inhibitor sildenafil^241^ and many other commonly used clinical drugs in UC are attributed to the inhibition of the NF‒κB pathway, which inhibits the expression of inflammatory cytokines and the infiltration of inflammatory cells, alleviating intestinal epithelial damage.

Vitamin D deficiency is one of the most common manifestations of malnutrition in UC patients.^13^ The vitamin D receptor plays a crucial role in regulating several physiological processes, such as cell growth, immune system function, calcium balance, and inflammation in the human body.^242^ The vitamin D/VDR axis has been shown to suppress the overexpression of HIF-1α in colonic epithelial cells by regulating the NF-κB pathway, thereby inhibiting the overexpression of inflammatory factors.^243^ The overexpression of VDR signaling can also maintain intestinal epithelial homeostasis and intestinal barrier function by upregulating claudin-2 and claudin-15.^244^

### The PI3K/AKT signaling pathway

**Fig. 4:**
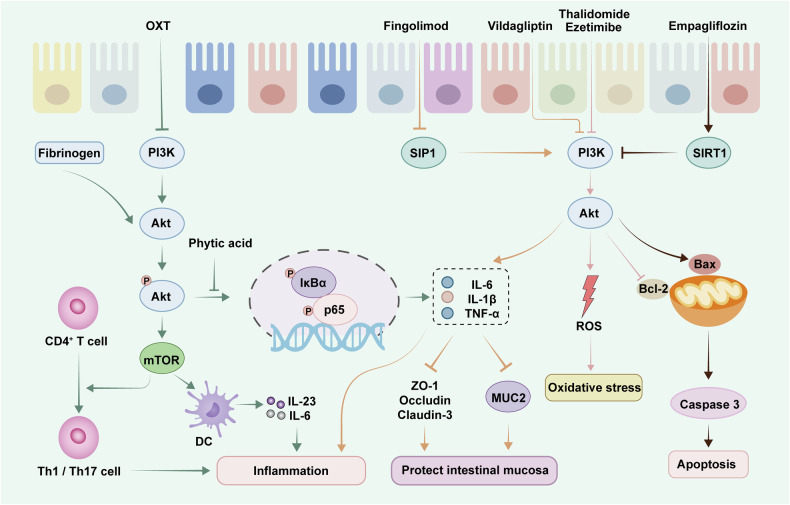
Key targets and upstream/downstream mediators of the PI3K-AKT signaling pathway in UC. Activation of the PI3K-AKT pathway can increase the phosphorylation level of the downstream signaling molecule mTOR and regulate the differentiation of DCs and T cells, which in turn affects the levels of inflammatory factors. It can also promote the secretion of inflammatory factors and regulate the expression of tight junction proteins in the intestinal mucosa by activating the NF-κB pathway. Furthermore, the PI3K-AKT pathway can regulate the expression of caspase-3, which in turn regulates the apoptosis of intestinal epithelial cells. The PI3K-AKT pathway also regulates the activation of intracellular ROS and modulates oxidative stress. In addition, fingolimod, vildagliptin, thalidomide, and other drugs can delay the disease progression of UC by modulating the PI3K‒AKT pathway. PI3K phosphatidylinositol-3-kinase-protein kinase B, Akt protein kinase B, SIRT1 sirtuin 1, SIP1 sphingosine-1-phosphate receptor, ROS reactive oxygen species

The PI3K/AKT pathway is an important cell signaling pathway that plays a key role in regulating various biological processes, such as cell proliferation, apoptosis, and metabolism.^245^ PI3K is a kinase that can be activated by a variety of external signals to catalyze the conversion of phosphatidylinositol diphosphate to phosphatidylinositol triphosphate. AKT, an effector molecule downstream of PI3K activation, is widely involved in the biological processes of the PI3K/AKT pathway.^246^ In recent years, increasing evidence has shown that the PI3K/AKT signaling pathway is closely associated with the occurrence and development of UC and is also an important signaling pathway for targeted UC therapy.^247,248^ The key targets of the PI3K/AKT pathway and their related regulatory mechanisms are shown in Fig. 4.

The results demonstrated that the expression of Muc2 in the colon of mice with aldo-keto reductase 1B10 deficiency is decreased, resulting in increased intestinal permeability; this is accompanied by the inhibition of AKT activity and an increase in AKT phosphorylation, which inhibits the expression of cytokines, resulting in intestinal immunodeficiency and increased rates of UC development.^249^ The neurosecretory hormone oxytocin (OXT) inhibits dendritic cell maturation, inhibits the expression of IL-23 and IL-6, and promotes the expression of TGF-β through the PI3K/AKT/mTOR pathway, thereby regulating intestinal immunity and improving UC symptoms.^250^ In inflammation-related studies, AKT has been demonstrated to be upstream of the NF-κB pathway. After phytic acid treatment, the phosphorylation level of AKT in UC mice was significantly inhibited. As a result, the release of IL-1β, IL-6 and TNF-α in the colon is reduced, the expression of tight junction proteins is increased, the integrity of the intestinal barrier is maintained, and the symptoms of UC mice are improved.^251^ The PI3K/AKT pathway can also ameliorate the colonic inflammatory phenotype and inhibit the activation of CD4^+^ T cells by suppressing the glycolytic pathway,^252^ and the activation of Akt signaling has been shown to promote mucosal healing.^253^

A variety of pharmacologic drugs that target the PI3K/AKT pathway have also shown efficacy in interventions against UC. Fingolimod, a sphingosine-1-phosphate receptor 1 modulator, regulates the immune response to UC by suppressing the AKT/mTOR pathway, thereby regulating the balance of proinflammatory cytokines and anti-inflammatory cytokines.^254^ Thalidomide is an immunomodulatory glutamate derivative that significantly reduces the protein expression levels of p-AKT and p-PI3K in intestinal epithelial cells and exerts anti-inflammatory effects by suppressing the activation of the PI3K/AKT pathway.^255^ Ezetimibe can also reduce the levels of NF-κB, TNF-α and IL-6 in the colorectal tissue of UC rats by suppressing the expression of AKT and p-AKT, thereby decreasing the infiltration of inflammatory cells and providing a favorable mucosal healing effect.^256^ In addition, the dipeptidyl peptidase IV (DPPIV) inhibitor vildagliptin inhibits intestinal epithelial apoptosis by suppressing the PI3K/AKT/NF-κB pathway.^257^ Disruption of the colonic barrier is the most common pathological change in animal models of UC, and studies have shown that the SGLT2 inhibitor empagliflozin can significantly increase the expression of SIRT-1, suppress the levels of PI3K and AKT, and increase the levels of tight junction proteins (claudin-1 and occludin), thereby improving the integrity of the colonic barrier.^258^ Interestingly, studies have demonstrated that some probiotics can also significantly improve intestinal inflammation symptoms through the PI3K/AKT signaling pathway.^259^

### The Wnt/β-catenin signaling pathway

**Fig. 5:**
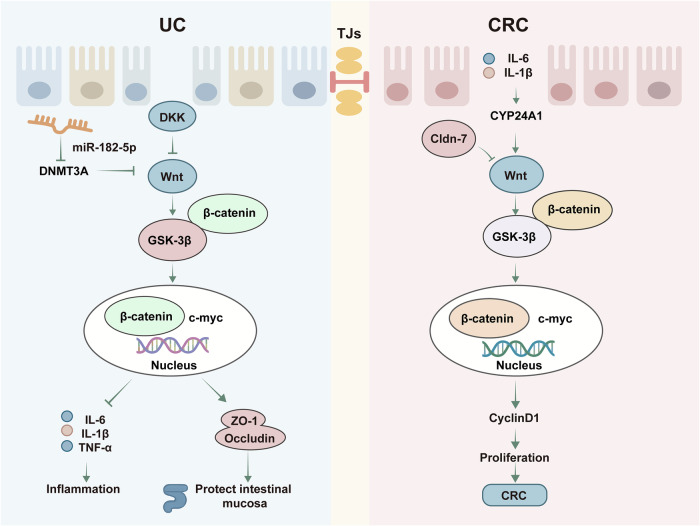
Key targets and upstream/downstream mediators of the Wnt/β‑catenin signaling pathway in UC. The Wnt/β-catenin signaling pathway regulates cell proliferation and differentiation and promotes the expression of intestinal mucosal tight junction proteins by regulating the expression of the serine/threonine kinase GSK-3β to protect the intestinal mucosa. The secreted protein DKK acts upstream of the Wnt/β-catenin pathway and regulates the proliferation of intestinal epithelial cells. The infiltration of many inflammatory cytokines and a lack of Cldn-7 can lead to abnormal activation of Wnt/β-catenin, which in turn causes the abnormal proliferation of intestinal epithelial cells and increases the expression of the oncogenic gene c-myc, which in turn significantly upregulates the expression of the cell cycle protein cyclin D1 and facilitates the transition from UC to CRC. DKK Dickkopf, GSK-3β glycogen synthase kinase-3β, DNMT3A DNA methyltransferase 3A, CYP24A1 cytochrome P450 family member 24A1, TJs tight junctions

The Wnt/β-catenin pathway regulates cell proliferation and differentiation and plays a crucial role in the pathogenesis of UC.^260^ In patients with UC, the Wnt/β-catenin pathway is often abnormally activated, resulting in abnormal proliferation and differentiation of intestinal mucosal epithelial cells, which in turn triggers an inflammatory response and tissue damage.^261,262^ Wnt, a key signaling molecule in the Wnt/β-catenin pathway, is also an important protein in the repair process of intestinal epithelial mucosal injury. It is required for colon healing and intestinal epithelial cell differentiation.^263,264^ Key targets of the Wnt/β-catenin pathway and its related regulatory mechanisms are shown in Fig. 5.

One study demonstrated that eicosapentaenoic acid promotes intestinal epithelial cell proliferation via the Wnt/β-catenin/c-myc pathway, thereby significantly improving UC symptoms.^265^ Additionally, activation of the Wnt/β-catenin signaling pathway has been shown to inhibit IL-1β-induced apoptosis of intestinal epithelial cells, increase the expression of ZO-1 and Occludin, improve the structure and function of the intestinal barrier, and reduce inflammatory cell infiltration.^266^ An additional study demonstrated that inhibition of the γ-aminobutyric acid receptor can promote the renewal and repair of the colonic epithelium in UC mice by regulating the Wnt/β-catenin signaling pathway.^267^ Claudin-7 (Cldn-7), a member of the tight junction protein family, is closely related to inflammation and tumor development, and Cldn-7 deficiency can significantly upregulate the expression of β-catenin and its downstream target genes c-myc and cyclin D1 by promoting aberrant activation of the Wnt/β-catenin pathway, which in turn leads to abnormal cell proliferation and tumorigenesis.^268^ Inflammatory cytokines (e.g., IL-6 and TNF-α) upregulate the expression of CYP24A1 in colon cancer cells by activating the NF-κB pathway, which in turn leads to abnormal activation of the Wnt/β-catenin pathway and promotes disease progression.^269^

### The AMPK signaling pathway

AMPK is a cellular energy sensor that responds to changes within cells to maintain energy balance and metabolic homeostasis.^270^ Once AMPK is activated, it affects cellular metabolism and function by regulating a variety of downstream targets. This has an important impact on overall metabolism and energy balance, making AMPK a target for the treatment of UC.^271^ The activation of AMPK has been shown to inhibit NF-κB pathway activation; reduce the plasma concentrations of IL-6, IL-1β, IFN-γ and TNF-α in UC mice; increase IL-10 levels; and alleviate the symptoms of UC.^272^ P2Y1R is a receptor that is preferentially activated by ADP and is significantly upregulated in the colonic epithelial cells of UC patients.^273^ Studies have shown that the inhibition of P2Y1R promotes the phosphorylation of epithelial AMPK; increases the expression of Claudin-1, ZO-1 and Occludin; and promotes the repair of damaged intestinal mucosa.^274^ Donepezil is an acetylcholinesterase inhibitor that promotes the activation of AMPK and inhibits the activation of the NF-κB pathway by facilitating the expression of low-density lipoprotein receptor-associated protein 1, thereby reducing inflammation and intestinal epithelial apoptosis in the colon.^275^ The SGLT2 inhibitor dapagliflozin has also been shown to reduce neutrophil infiltration in colon tissue and significantly improve the histological symptoms of UC rats through the AMPK/NF-κB axis.^276^ The commonly used hypoglycemic drug metformin is an additional AMPK activator that can inhibit the release of inflammatory cytokines by activating the AMPK pathway, thus exerting an obvious anti-inflammatory effect.^277,278^

### TLR signaling pathway

**Fig. 6:**
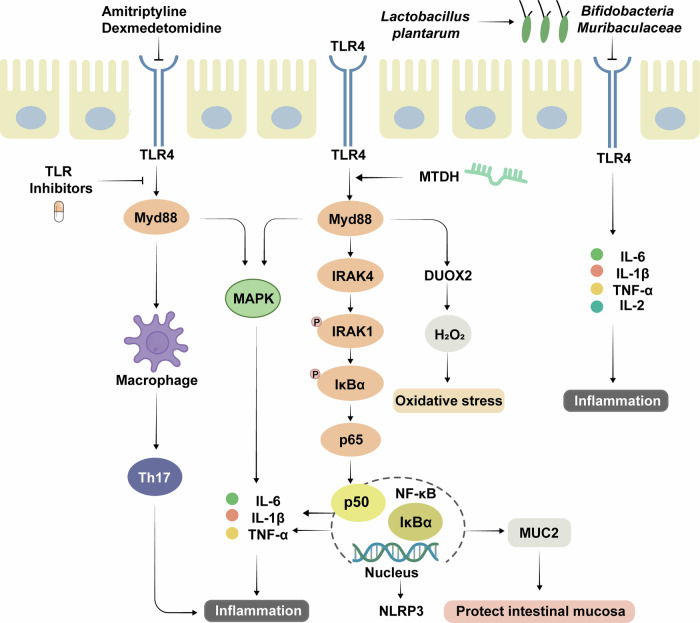
Key targets and upstream/downstream mediators of the TLR signaling pathway in UC. Activation of the TLR pathway is essential for the normal function of the immune system. The TLR signaling pathway is activated by the engagement of the TLR receptor to form a dimer, followed by the activation of the downstream IRAK kinase family through the recruitment of Myd88, which promotes the expression of NF-κB and MAPK-related proteins, thereby regulating the secretion of inflammatory factors. In addition, it can play an anti-inflammatory role by activating macrophage polarization and regulating the activity of Th17 cells. The application of TLR inhibitors can reduce the expression of inflammatory factors and the level of H_2_O_2_, thus suppressing the inflammatory response and oxidative stress. Moreover, the TLR signaling pathway can regulate the gut microbiota and thus treat UC. TLR4 Toll-like receptor 4, Myd88 Myeloid Differentiation Primary Response Gene 88, MTDH Metadherin, IRAK4 Interleukin-1 Receptor Associated Kinase 4, DUOX2 Dual Oxidase 2, NLRP6 Nucleotide-binding oligomerization domain-like receptor protein 6

TLRs are a class of receptor proteins that play a key role in the immune system by recognizing foreign pathogens such as bacteria and viruses through specific molecular patterns that trigger immune responses.^279^ Activation of the TLR pathway involves a series of molecular events. When the TLR receptor binds to its ligand, it activates downstream signaling pathways that trigger the production of a variety of signaling molecules and cytokines, which ultimately leads to an inflammatory response and the activation of immune cells.^280^ Therefore, researchers are exploring the use of the TLR pathway as a new way to treat UC.^281^ The key targets of the TLR pathway and their related regulatory mechanisms are shown in Fig. 6.

Activation of the TLR pathway is essential for the normal function of the immune system, but overactivation of this pathway has occurred in UC patients and UC animal models. Inhibition of TLR signaling can effectively alleviate the symptoms of colitis, colon tissue damage and mucosal inflammation; inhibit the expression of TNF-α and IL-1β; and prevent the progression of UC to colon cancer.^282,283^ TLR4 is one of the most important and widely involved members of the TLR family, and it is also a key signaling molecule in the treatment of UC by targeting the TLR pathway.^284^ When TLR4 binds to its ligand, it activates Myd88, which triggers a series of downstream signaling events, including the activation of the NF-κB and MAPK pathways and the production of inflammatory cytokines such as IL-6 and TNF-α. By blocking TLR4/Myd88 signaling, the number of goblet cells in the intestinal mucosa can be restored, and the expression of tight junction proteins can be increased, thereby restoring the structure and function of the intestinal barrier.^285–287^ The MAPK pathway is an important pathway related to UC,^288^ and its inhibition reduces inflammation by downregulating the levels of proinflammatory cytokines.^289^ Irisin exerts anti-inflammatory effects by inhibiting the MAPK pathway in RAW264.7 macrophages.^290^ Both the α2-adrenergic agonist dexmedetomidine and the tricyclic antidepressant amitriptyline have been found to improve UC by inhibiting TLR4.^291,292^ Furthermore, the probiotic *Lactobacillus plantarum* has been reported to ameliorate UC by modulating the TLR signaling pathway.^293^

Interleukin-1 receptor-associated kinase 1/4 (IRAK1/4) is the main kinase in the TLR-mediated pathway, and inhibition of IRAK1/4 can block the TLR4/NF-κB pathway and reduce intestinal inflammation. Furthermore, inhibition of IRAK1/4 can increase the expression of tight junction proteins and mucin mRNAs and maintain the integrity of the intestinal barrier.^294^ High expression of metadherin (MTDH) positively regulates the TLR-induced inflammatory response and promotes the secretion of inflammatory cytokines; however, MTDH deletion suppresses inflammatory cytokines in TLR-induced macrophages.^295^ In addition, high expression of TLR4 can increase H_2_O_2_ production in the colonic mucosa of UC mice, induce redox activity in the mucosa, exacerbate oxidative stress, and promote the disease progression of UC.^296^ NLRP3 is a core member of the inflammasome and a multiprotein complex that regulates inflammatory responses downstream of TLRs.^297^ Anemoside B4 attenuates colitis symptoms by suppressing the activation of NLRP3 and the phosphorylation of MLC2, which in turn regulates the dysbiosis of the gut microbiota.^298^

### The JAK/STAT signaling pathway

**Fig. 7:**
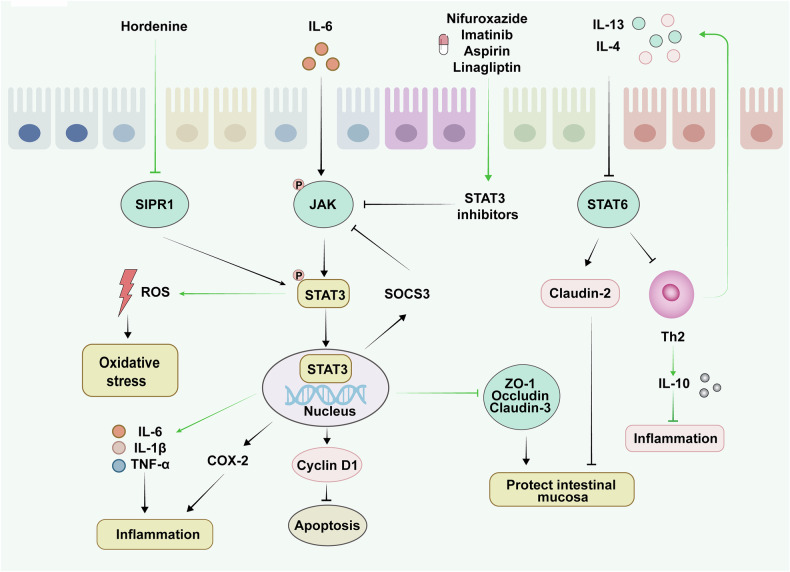
Key targets and upstream/downstream mediators of the STAT signaling pathway in UC. The STAT pathway regulates cell growth and immune responses. When cells are specifically stimulated by the inflammatory factor IL-6, the downstream JAK signaling pathway is activated, leading to the phosphorylation of STAT3, which in turn regulates the levels of inflammatory factors as well as tight junction proteins and mitigates UC. In addition, it can also inhibit oxidative stress by suppressing ROS signaling while regulating the expression of cell cycle proteins and thereby modulating apoptosis in intestinal epithelial cells. Similarly, when intestinal epithelial cells are stimulated with IL-4 and IL-13, STAT6 is phosphorylated. This phosphorylation inhibits claudin-2 expression and protects the structure and function of the intestinal mucosa while promoting the expression of IL-10 to inhibit inflammatory responses. SIPR1 sphingosine-1-phosphate receptor 1, JAK Janus kinase, STAT3 signal transducer and activator of transcription 3, STAT6 signal transducer and activator of transcription 6, SOCS3 suppressor of cytokine signaling 3

The signal transducer and activator of transcription (STAT) signaling pathway is involved in the control of vital biological processes such as cell growth, differentiation and the immune response.^299^ Under normal conditions, the STAT protein is in an inactive state. When cells are specifically stimulated, ligands bind to receptors after being activated. This activation triggers downstream signaling pathways, leading to the phosphorylation of STAT proteins, thereby regulating the expression of a series of genes and affecting cell function and physiological activity.^300^ Several studies in recent years have suggested that inhibition of the STAT pathway may be beneficial in the treatment of UC.^301,302^ Key targets of the STAT pathway and their related regulatory mechanisms are shown in Fig. 7.

STAT3 is a key member of the STAT protein family, and its high expression promotes the excessive secretion of inflammatory cytokines and activates CD4^+^ T cells to aggravate the intestinal inflammatory response.^303^ Nifuroxazide, a potent STAT3 inhibitor, directly inhibits STAT3 to reduce the inflammatory response of the colon and promote intestinal epithelial barrier healing.^304^ Studies have shown that hordenine decreases the expression of proinflammatory cytokines by suppressing the S1P/S1PR1/STAT3 pathway and promotes the healing of colonic ulcers by regulating the expression of tight junction proteins, including occludin and ZO-1.^305^ Increasing the level of IL-22 in UC mouse models promotes mucosal barrier defense and repair by regulating the expression of STAT3.^306^ Studies have also confirmed that the aminosalicylic acid preparation mesalazine inhibits the levels of proinflammatory cytokines such as TNF-α and IL-13 through the IL-6/STAT3 signaling pathway, restoring normal levels of ZO-1 and IL-10 in the colon and improving UC progression in rats.^307^ The JAK/STAT3 signaling pathway, one of the most important modes of STAT signaling, regulates cell function through cytokine-receptor interactions, activation of the JAK protein kinase, and phosphorylation of the STAT protein. Inhibition of the JAK/STAT3 pathway is an important strategy for improving UC.^308,309^ Drugs such as imatinib, aspirin, and linagliptin have been shown to decrease inflammation and improve the symptoms of UC by inhibiting the JAK/STAT3 signaling pathway, decreasing inflammatory cell infiltration and promoting intestinal mucosal repair via the regulation of immune responses.^310–312^

STAT6 is another important member of the STAT protein family that is involved in the regulation of the Th2 immune response, and the signal transduction of the Th2 cytokines IL-4 and IL-13 is closely associated with the pathogenesis of UC.^313^ High expression of claudin-2, a tight junction protein, can increase the permeability of the intestinal barrier and disrupt its structure. In animal experiments, STAT6 inhibition reduced the expression of claudin-2, protected the structure and function of the intestinal barrier, and alleviated the symptoms of UC.^314^ Further research on the mechanism of action of STAT6 in the treatment of UC is needed to obtain a comprehensive understanding of the therapeutic potential of STAT6.

### LncRNAs

**Fig. 8:**
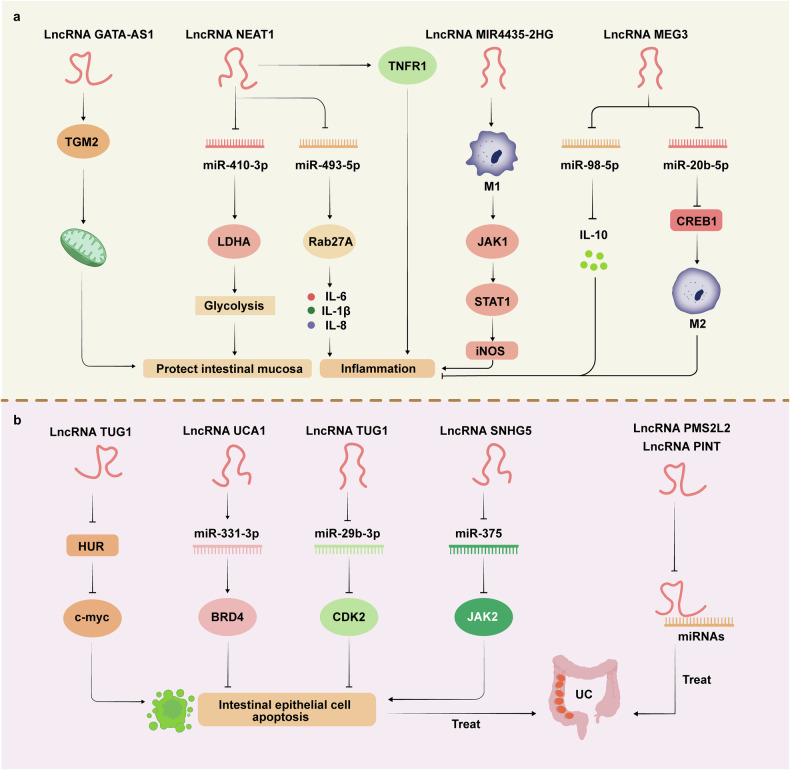
Targeted therapeutic effects of lncRNAs in UC. LncRNAs play important regulatory functions in cells by regulating gene expression, inflammatory signaling, and many other pathways. **a**, **b** A variety of lncRNAs play critical roles in the development of UC, including in the regulation of mitochondrial function, the inhibition of inflammatory factors via the modulation of inflammation-related pathways, the protection of the intestinal mucosa via the regulation of tight junction protein expression, and the inhibition of the apoptosis of intestinal epithelial cells via the sponging of miRNAs. These activities of lncRNAs can regulate disease progression in UC. LDHA lactate dehydrogenase A, TNFR1 tumor necrosis factor receptor 1, Rab27A Ras-related protein Rab-27A, CREB1 cAMP responsive element binding protein 1, BRD4 bromodomain-containing protein 4, CDK2 cyclin-dependent kinase 2, JAK2 Janus kinase 2

Long noncoding RNAs (lncRNAs) are a class of RNA molecules that are more than 200 nucleotides in length and play important roles in cells by participating in the regulation of biological processes such as gene expression, substrate transport, and signal transduction.^315,316^ In UC, studies have shown that specific lncRNAs may be involved in the pathogenesis of the disease and that targeting them may be a valuable approach for treatment.^317^ LncRNAs are involved in the regulation of multiple UC disease-related processes, as shown in Fig. 8.

The lncRNA UCA1 promotes the progression of UC through the miR-331-3p/BRD4 molecular axis. However, deletion of lncRNA UCA1 decreases the levels of TNF-α, IL-6 and IL-1β and reduces the apoptosis of intestinal epithelial cells, suggesting that knockdown of lncRNA UCA1 may alleviate the symptoms of UC.^318^ In the UC mouse model, the lncRNA TUG1 positively regulates the HuR/c-myc axis through interaction with HuR, resulting in the upregulation of c-myc expression. Additionally, the lncRNA TUG1 negatively regulates the miR-29b-3p/CDK2 signaling pathway by binding to miR-29b-3p, resulting in the downregulation of CDK2 expression, which ultimately inhibits intestinal epithelial apoptosis.^319^ As a competitive endogenous RNA (ceRNA), the lncRNA SNHG5 regulates intestinal epithelial cell proliferation and apoptosis through the miR-375/JAK2 axis, and the knockdown of the lncRNA SNHG5 significantly improves the dysfunction of intestinal epithelial cells.^320^ In addition, studies have confirmed that both the lncRNA PMS2L2 and the lncRNA PINT can regulate the disease progression of UC by adsorbing microRNAs.^321,322^

The lncRNA GATA6-AS1 has been shown to interact with transglutaminase 2 (TGM2), resulting in changes in mitochondrial function, promoting the restoration of damaged intestinal mucosal epithelial function and mucosal healing, and improving the prognosis of UC.^323^ The lncRNA NEAT1 is highly expressed in UC patients, and in vitro experiments have shown that it regulates its downstream target LDHA by adsorbing miR-410-3p, thereby inhibiting the glycolysis pathway and improving intestinal epithelial cell dysfunction.^324^ Inhibition of the lncRNA MIR4435-2HG regulates macrophage M1/M2 polarization through the JAK1/STAT1 pathway, thus alleviating intestinal inflammation and colon injury.^325^

In UC rats, the expression of the lncRNA MEG3 was significantly reduced. When the expression of the lncRNA MEG3 was exogenously increased, it promoted the expression of IL-10 by sponging miR-98-5p, thereby alleviating colon ulcers in UC rats.^326^ The lncRNA MEG3 can also competitively bind to miR-20b-5p. As a result, it promotes CREB1 transcription and M2 macrophage polarization. This, in turn, reduces inflammatory cell infiltration and alleviates colon injury.^327^ QRT‒PCR analysis also demonstrated that Lnc78583 overexpression upregulated HOXB13 and IL-10 to inhibit inflammation by inhibiting the binding of miR3202 to HOXB13, indicating that Lnc78583 has a protective effect on lipopolysaccharide-induced UC inflammation by targeting HOXB13.^328^

### miRNAs

**Fig. 9:**
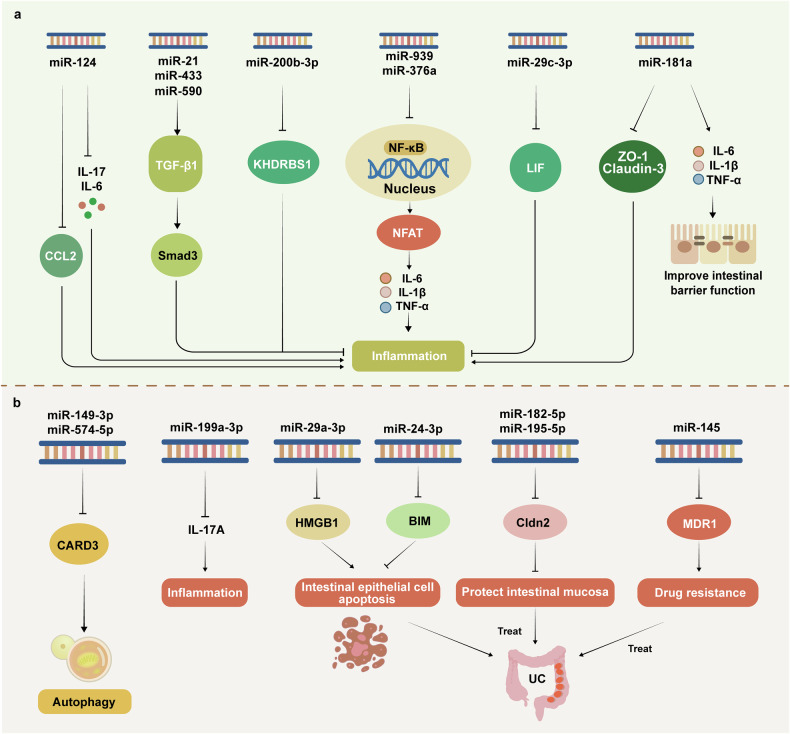
miRNAs are important for the targeted treatment of UC. miRNAs play important roles in regulating gene expression in cells by binding to the mRNAs of target genes and inhibiting their translation or promoting their degradation, thus regulating the expression of sets of relevant genes. miRNAs can be used as tissue markers for UC patients and may be useful for the diagnosis of UC. **a**, **b** In terms of therapy, miRNAs can inhibit inflammation-related signaling pathways and thus inhibit the levels of inflammatory factors and increase the expression of tight junction proteins to improve the permeability of the intestinal barrier. miRNAs can also increase the propagation of beneficial bacteria in the intestinal tract, suppress the proliferation of harmful bacteria, and maintain the stability of the intestinal microbiota. Moreover, miRNAs can improve the drug sensitivity of UC therapeutic drugs by regulating drug resistance genes. TGF-β1 transforming growth factor-beta 1, Smad3 SMAD family member 3, CCL2 C-C motif ligand 2, KHDRBS1 KH domain containing, RNA binding, signal transduction associated 1, NFAT nuclear factor of activated T cells, LIF leukemia inhibitory factor‌, MDR1 multidrug resistance 1, Cldn2 claudin 2‌, HMGB, high mobility group protein B1, MCL-1 myeloid cell leukemia-1

microRNAs (miRNAs) are a class of short, noncoding RNA molecules, usually approximately 20–25 nucleotides in length, that play important roles in cells by regulating gene expression.^329^ By binding to the mRNAs of target genes, miRNAs can inhibit their translation into proteins or promote their degradation, thereby regulating the expression of sets of related genes.^330^ Studies have shown that the modulation of miRNA expression can alleviate the symptoms of UC via in vitro and in vivo experiments, which provides hope for the application of miRNAs in UC treatment.^331,332^ The mechanisms by which miRNAs are involved in the regulation of UC are shown in Fig. 9.

MiR-21, miR-433 and miR-590 have been shown to be useful tissue markers in UC patients. The overexpression of miR-21 and miR-433 and the downregulation of miR-590 in UC patients are significantly related to TGF-β signaling pathway activity and can be used as potential targets for UC treatment.^333^ The expression of miR-124 is low in UC patients, and in vitro experiments have shown that the upregulation of miR-124 can promote a decrease in proinflammatory cytokines, including IL-17 and IL-6, as well as the chemokine CCL2, which plays a role in alleviating UC.^334^ MiR-200b-3p attenuates TNF-α-induced inflammation and apoptosis in intestinal epithelial cells and slows the progression of UC by targeting KHDRBS1.^335^ Moreover, miR-939 and miR-376a inhibit the activation of NF-κB and NFAT, which in turn inhibits the expression of inflammatory factors, leading to partial restoration of the intestinal barrier structure and function in UC rats.^336^ In addition, miR-145 regulates the expression of the drug resistance gene MDR1, thereby improving drug sensitivity to 5-aminosalicylic acid and glucocorticoids in the treatment of UC.^337^

During the occurrence and progression of UC, repair of the intestinal mucosal barrier acts as a marker of UC remission.^338,339^ High expression of miR-24-3p, miR-199a-3p and miR-29a-3p has been shown to improve the intestinal barrier in UC, thereby alleviating disease progression.^340–342^ The increased expression of miR-195-5p also significantly reduces the expression of Cldn2, increases the expression of tight junction proteins, and increases the permeability of the intestinal barrier.^343,344^ Dual luciferase reporter assays confirmed that miR-330 negatively regulates the expression of IRAK1 in colonic tissues, alleviates intestinal mucosal injury, and inhibits proinflammatory factors.^345^ Conversely, low expression of miR-29c-3p in UC has been shown to promote the exacerbation of the inflammatory response.^346^

High expression of miRNA-182-5p increases the permeability of the intestinal barrier and accelerates destruction of the intestinal barrier by suppressing the expression of Claudin-2.^347^ MiR-181a increases the abundance of beneficial bacteria, inhibits the expression of the inflammatory cytokines IL-6, TNF-α and IL-1β, upregulates the expression of claudin-1 and ZO-1, and improves intestinal barrier function by regulating the composition of the intestinal microbiota.^348^ Studies have also shown that two types of harmful bacteria in the gut, *Fusobacterium nucleatum* and *Bacteroides fragilis*, promote the proliferation of UC intestinal inflammatory cells by regulating miR-574-5p and miR-149-3p, respectively, which disrupts the integrity of the intestinal epithelial barrier.^349,350^

## Novel cellular processes and pharmacological targets

Although many studies have verified the molecular mechanisms and related drug targets involved in the process of UC occurrence and development, many new insights have been proposed into the pathogenesis of UC in recent years. These new insights may provide new personalized diagnosis and treatment options for the future treatment of UC. In this section, we discuss new potential approaches for UC therapy that are less widely recognized and hold great promise for future research.

### Ferroptosis

**Fig. 10:**
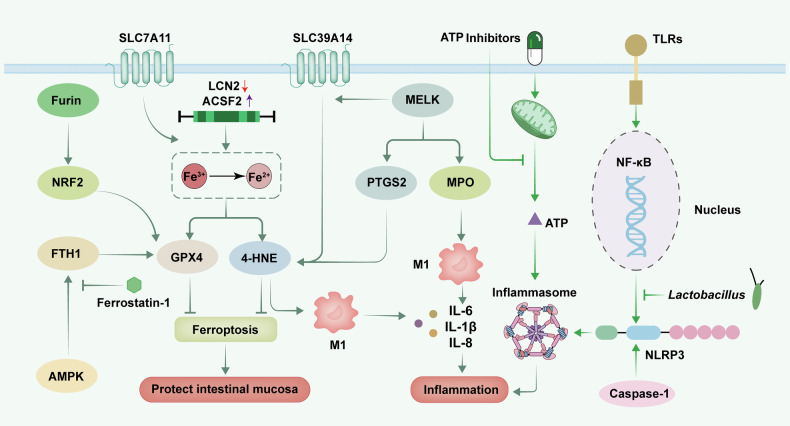
Targeted regulation of UC by ferroptosis and the inflammasome. Ferroptosis is a mode of cell death caused by the excessive accumulation of lipid peroxides due to disrupted intracellular metabolic pathways. It is distinct from apoptosis and is closely related to intracellular iron metabolism and lipid homeostasis. Ferroptosis is accompanied by alterations in iron death-related genes (LCN2 and ACSF2) as well as downregulation of GPX4 and upregulation of the expression of the lipid peroxidation marker 4-HNE. After the inhibition of ferroptosis, the expression of these proteins is significantly reversed, and damage to the intestinal mucosa is significantly reduced. MELK inhibits ferroptosis-related genes, reduces the secretion of inflammatory factors, and attenuates the intestinal inflammatory response by inhibiting M1 macrophage polarization. Moreover, activation of both the NRF2 pathway and the AMPK pathway inhibits the occurrence of ferroptosis, which in turn ameliorates intestinal epithelial injury. Inflammasomes are important for immune regulation and generally include apoptosis-associated microsomal proteins (ASCs), caspase proteases, and NOD-like receptor family proteins (e.g., NLRP3). The inhibition of inflammasome complex formation effectively reduces the release of IL-1β and IL-18 and the associated inflammatory injury in intestinal epithelial cells. The inhibition of mitochondrial ATP synthesis can also inhibit the formation of inflammasomes. SLC7A11 Solute Carrier Family 7 Member 11, NRF2 Nuclear Factor Erythroid 2-Related Factor 2, GPX4 Glutathione Peroxidase 4, FTH1 Ferritin Heavy Polypeptide 1, LCN2 Lipocalin-2, ACSF2 Acyl CoA synthetase 2, 4-HNE 4-Hydroxynonenal, MELK Maternal Embryonic Leucine Zipper Kinase, PTGS2 Prostaglandin-endoperoxide synthase 2, NLRP3 NOD-like receptor thermal protein domain associated protein 3

Ferroptosis is a recently discovered programmed cell death modality associated with intracellular iron overload that is distinct from apoptosis.^351^ In the process of ferroptosis, cells are affected by oxidative stress and lipid peroxidation, resulting in the rupture of cell membranes and cell death, which is manifested mainly by the reduction in GPX4, the core enzyme of the antioxidant system (glutathione system), and the high expression of the lipid peroxidation marker 4-HNE.^352^ In LPS/IFN-γ-induced inflammation models, lipid peroxidation levels are increased, and GPX4 expression is significantly reduced, promoting ferroptosis in intestinal epithelial cells.^353^ These findings imply that ferroptosis is involved in the occurrence of UC. In recent years, it has been proposed that UC can be treated by inhibiting ferroptosis to reduce intestinal epithelial cell damage and protect the structure and function of the intestinal barrier.^354,355^ The molecular mechanism by which ferroptosis is involved in the occurrence and development of UC is shown in Fig. 10.

The highly expressed protein convertase Furin inhibits intestinal epithelial cell damage in a rat model of UC by activating the Nrf2‒Gpx4 pathway, thus alleviating the symptoms of UC.^356,357^ The activated AMPK pathway can upregulate the expression of FTH1 and GPX4 in intestinal tissues and can improve iron metabolism and redox disorders in the colons of UC rats, thereby effectively treating UC.^358^ MELK, a serine/threonine kinase, is a member of the AMPK-related kinase family.^359^ The inhibition of MELK expression significantly downregulates the expression of the ferroptosis-related genes PTGS2 and SLC39A14 and significantly reduces the expression of the lipid peroxidation marker 4-HNE and the oxidative stress marker MPO, thereby inhibiting macrophage infiltration and M1 polarization, reducing the secretion of proinflammatory factors, and alleviating intestinal tissue damage and intestinal inflammation.^360^ The application of ferroptosis inhibitors also significantly upregulates the expression of the ferroptosis-related gene ACSF2, which further demonstrates that the inhibition of ferroptosis can maintain the stability of the intestinal barrier.^361^ Interestingly, in a 2.5% dextran sodium sulfate-induced mouse model of colitis, mice fed a high-fat diet presented significantly reduced expression of markers of lipid peroxidation and ferroptosis in the gut in a manner independent of obesity, protecting the gut from inflammatory damage by inhibiting ferroptosis.^362^ Although ferroptosis is not widely studied in the treatment of UC, it will certainly play a greater role in future research on the treatment of UC and the delay of disease progression.

### Inflammasomes

Inflammasomes are multiprotein complexes that were first proposed in 2002.^363^ Their formation is related to the immune system’s fight against infection and the maintenance of tissue homeostasis. Inflammasomes promote the maturation and secretion of the cytokine precursors pro-IL-1β and pro-IL-18 in the process of innate immune defense by inducing the activation of caspase-1, thereby triggering an inflammatory response.^364^ UC plays a critical role in inflammation and immune regulation.^365,366^ The molecular mechanisms by which inflammasomes are involved in the occurrence and development of UC are shown in Fig. 10.

As a vital component of innate immunity, the NLRP3 inflammasome, which is the most common inflammasome, plays a crucial role in the body’s immune response and the occurrence of UC.^367^ NLRP3 is a key component of the NOD-like receptor family of intracellular sensors. Given its importance in the immune system, the NLRP3 inflammasome has drawn significant attention in the study of UC. Deubiquitination of active NLRP3 triggers ASC aggregation and the recruitment of precaspase-1, which leads to the formation of the NLRP3 inflammasome and the release of proinflammatory cytokines such as IL-1β and IL-18.^368–370^ HSF2 and DLG2 can block the oligomerization of ASC by interfering with the interaction between ASC and NLRP3, thereby inhibiting the activation of the NLRP3 inflammasome and the upregulation of IL-1β expression and alleviating the inflammatory response.^371,372^ Atranorin directly binds to ASC and subsequently inhibits ASC aggregation and the formation of NLRP3 inflammasome complexes, thereby reducing the release of IL-1β and IL-18.^373^ Moreover, ferulic acid and *Lactobacillus* have been confirmed to inhibit the formation of the NLRP3 inflammasome by inhibiting TXNIP, which reduces the inflammatory damage to UC intestinal epithelial cells.^374–376^ *Bacteroides dorei* BDX-01 downregulates the expression of NLRP3, ASC and Caspase-1 in colon tissues and reduces the secretion of IL-1β by regulating bile acid metabolism pathways.^377^ Interestingly, prebiotic supplementation has also been demonstrated to inhibit the formation of inflammasomes, thereby alleviating symptoms and disease progression in UC.^378,379^ It has also been proposed that the formation of inflammasomes can be inhibited by inhibiting the synthesis of adenosine triphosphate (ATP).^380^ Overall, these results suggest that the symptoms of UC could be alleviated by regulating the formation and activation of inflammasomes and reducing the release of inflammatory mediators.^381,382^

### Th/Treg cell balance

Th/Treg homeostasis refers to the homeostatic relationship between regulatory T (Treg) and helper T (Th) cells.^383^ Treg cells play a critical role in the immune system by suppressing the immune response and maintaining immune tolerance, whereas Th cells are involved mainly in the regulation and promotion of the immune response.^384^ Maintenance of the Th/Treg balance is essential for proper immune system function. In UC and other inflammatory diseases, the Th/Treg balance is disrupted, usually resulting in increased activity or increased numbers of Th17 cells and decreased function or a decreased number of Treg cells.^385–387^ Therefore, researchers are interested in regulating Th/Treg homeostasis and its role in the occurrence and progression of UC.

Bone marrow mesenchymal stem cells that silence PD-L1 can upregulate the expression of PTEN, inhibit the Akt/mTOR pathway, induce Treg differentiation, and inhibit intestinal inflammation.^388^ Knockout of α2,6-sialyltransferase suppresses the polarization of CD4^+^ T cells to Th17 cells while promoting their polarization to Treg cells, inhibiting the secretion of IL-17a, and alleviating UC symptoms.^389^ GSK-J1 is a specific JMJD3 inhibitor^390^ that can affect the demethylation of histone H3K27me3, regulate Th17/Treg cell differentiation, suppress the secretion of inflammatory factors, and improve the intestinal mucosal immune dysfunction caused by DSS in experimental acute UC model mice.^391^ *Lactobacillus paracasei* R3 and *Bacteroides faecis* can both restore the integrity of the gut barrier by affecting Th17/Treg homeostasis, which in turn increases the expression of tight junction proteins and regulates the gut microbiota.^392^ Therefore, regulating the Th/Treg balance can significantly alleviate the intestinal inflammatory response and protect the intestinal barrier. Strategies to maintain the Th/Treg balance in the intestinal mucosa may be effective treatment approaches for UC.^393^

### Other novel targets

P2Y14R, a member of the purinergic receptor family, has been shown to promote the transcription of RIPK1 through the cAMP/PKA/CREB signaling axis, thereby driving necrotic apoptosis of intestinal epithelial cells and exacerbating intestinal barrier disruption and inflammatory responses.^394^ P2Y6R, which also belongs to the purinergic receptor family, is involved in inflammatory responses and immune regulation by regulating the signaling of extracellular nucleotides (e.g., UDPs) and effectively ameliorates the effects of DSS-induced ulcerative colitis in mice.^395^ TYK2 is a tyrosine kinase that plays a key role in the cytokine signaling pathway. The inhibition of TYK2 can reduce the production of proinflammatory cytokines, thereby alleviating the inflammatory response in UC.^396,397^

## Interventions for Ulcerative colitis

The progression of UC is a complex process that is attributed primarily to genetics and the environment and secondarily to a disturbance of the gut microbiota,^398,399^ abnormal immunoregulation,^400^ and psychological stress. Owing to the variety of contributing factors and prolonged nature of UC, the development of new clinical drugs and novel personalized treatments are research priorities.^401,402^ Herein, we describe lifestyle interventions that have been shown to improve UC. Furthermore, we systematically summarize the options for pharmacological and herbal interventions, including those in clinical trials.

### Lifestyle interventions

The occurrence and development of UC are often closely related to individual lifestyle habits, and lifestyle interventions (e.g., dietary changes) can help control the progression of the disease and improve the prognosis.^403,404^ Dietary therapy has gradually become one of the main treatments for patients with early-stage UC.^405^ Specifically, both individualized diets and intermittent fasting have demonstrated good intervention effects on this disease.^406,407^ UC patients are prone to malnutrition due to insufficient dietary intake, and nutritional supplementation is recommended during treatment.^408^ The recommended dietary supplements for UC patients include fat,^409^ protein^410^ and vitamins.^411,412^ Fermented foods have been recommended for UC patients.^413,414^ Additionally, probiotics have been used to regulate imbalances in patients’ intestinal flora.^415–417^

The data of animal experiments show that increased milk intake significantly improves inflammatory cell infiltration in the intestinal mucosa and ameliorates intestinal ulcers in UC mice, with the effect of regulating the intestinal microbiota.^418,419^ Studies have also shown that polyphenols in tea significantly alleviate UC by preventing the destruction of colorectal intestinal structure and function and inhibiting the expression of inflammatory factors.^420,421^ Patients are advised to reduce excessive intake of acidic foods to prevent exacerbation of UC symptoms.

In addition to dietary adjustments, other lifestyle factors also play a role in managing UC. Moderate exercise has been shown to have a beneficial effect on people with UC, with improvements in overall physical health, including boosting the immune system, reducing stress, improving mental health, and increasing muscle strength.^422^ The results also confirmed that exercise can improve clinical symptoms and colon inflammation in UC mice by balancing oxidative stress.^423^

### Pharmacological and herbal interventions

Although lifestyle interventions can alleviate the symptoms of UC patients in the early stage, symptoms in the later stage cannot be treated by simple lifestyle interventions alone.^424^ Therefore, we review the drugs commonly used in UC treatment and those currently in clinical trials (Table 1). At present, the treatment options for UC mainly include Western medicine, traditional Chinese medicine (TCM), and surgical treatment.^425–428^ Low-dose γ radiation has also been suggested to be beneficial for UC treatment.^429^ Western medicines are most commonly used in clinical practice and include aminosalicylic acid preparations,^430,431^ corticosteroids,^432^ and biologics.^433,434^ In addition, melatonin, vitamins, prebiotics and other adjuvants have been used to treat UC.^435^ TCM treatment is based on Chinese herbal compounds and decoctions^436^ and includes external treatments such as acupuncture.^437–439^

**Table 1:** The drugs currently in clinical trials and animal experiments for UC treatment

Class	Drug name	Experimental models	Registered clinical trails	Outcome	References
Aminosalicylic acid preparations	Mesalazine	UC patients	Phase 1	Rectal bleeding↓ Frequency of defecation↓	^442^
	Mesalazine	UC patients	Phase 3	Fecal calreticulin↓	^443^
	Mesalazine	Male albino rats	Animal experiment	STAT3, IL-6, IL-13 ↓ IL-10, ZO-1↑	^307^
	Olsalazine	Male BALB/c mice	Animal experiment	TNF-α, IFN-γ, IL-1β, IL-17 ↓ IL-2, IL-22, IL-10↑	^430^
	5-ASA	Male Wistar rats	Animal experiment	CAI, CDS, MDA↓ GSH↑	^431^
	5-ASA	SPF C57BL/6 WT mice	Animal experiment	IL-10, IL-22, TGF-β ↑ TNF-α ↓	^440^
	Sulfasalazine	UC patients	Clinical trails	Rectal bleeding↓ Frequency of defecation↓	^444–447^
	Sulfasalazine	Male Wister rats	Animal experiment	NF-κB, TNF-α, IL-6, IFN-γ, IL-1β ↓	^238^
Corticosteroid	Budesonide	UC patients	Phase 3	Rectal bleeding↓ Frequency of defecation↓	^452,454^
	Dexamethasone	UC patients	Phase 2	Rectal bleeding↓ Frequency of defecation↓	^456^
	Dexamethasone	Bioinformatics analysis	——	Rectal bleeding↓ Frequency of defecation↓	^457^
	Azithromycin	Male albino Wistar rats	Animal experiment	iNOS, NF-κB, TNFα, IL-1β, IL-6 ↓ IL-10↑	^125^
	Buspirone	Male Wistar rats	Animal experiment	TNF-α, MPO↓	^126^
Bio-formulation	Tofacitinib	UC patients	Phase 3	Rectal bleeding↓ Frequency of defecation↓	^467,468,470^
	Tofacitinib	UC patients	Phase 2	Adverse reaction↓	^469^
	Upadacitinib	UC patients	Phase 3	Rectal bleeding↓ Frequency of defecation↓	^474^
	Upadacitinib	UC patients	Phase 2b	Rectal bleeding↓ Stomachache↓	^475^
	Upadacitinib	UC patients	Phase 2b/Phase 3	Adverse reaction↓	^476,477^
	Upadacitinib	UC patients	Phase 3	Rectal bleeding↓ Frequency of defecation↓ Adverse reaction↓	^478–480^
	Filgotinib	UC patients	Phase 2b/3	Rectal bleeding↓ Stomachache↓	^481^
	Filgotinib	UC patients	Phase 2b/3	Frequency of defecation↓ Adverse reaction↓	^482^
	Filgotinib	UC patients	Phase 2b/3	Rectal bleeding↓ Frequency of defecation↓ Adverse reaction↓	^483–485^
	Filgotinib	UC patients	Phase 2b/3	Rectal bleeding↓ Frequency of defecation↓	^486–488^
	Mirikizumab	UC patients	Phase 2 /Phase 3	IL-1β, IL-6, CXCL9, CXCL10↓	^492^
	Mirikizumab	UC patients	Phase 2	Rectal bleeding↓ Frequency of defecation↓ Adverse reaction↓	^493^
	Mirikizumab	UC patients	Phase 2 /Phase 3	Rectal bleeding↓ Frequency of defecation↓	^494–496^
	Mirikizumab	UC patients	Phase 3	Fecal calreticulin↓ CRP↓	^497^
	Mirikizumab	UC patients	Phase 2	Rectal bleeding↓ Frequency of defecation↓	^498^
	Ustekinumab	UC patients	Phase 3	Rectal bleeding↓ Frequency of defecation↓ Adverse reaction↓	^501,502^
	Ustekinumab	UC patients	Phase 2/3	Rectal bleeding↓ Adverse reaction↓	^503,504^
	Ustekinumab	UC patients	Phase 3	Rectal bleeding↓ Frequency of defecation↓ Adverse reaction↓	^505–507^
	Infliximab	UC patients	Phase 1 /Phase 3	Rectal bleeding↓ Endoscopic mitigation	^509–511^
	Infliximab	UC patients	Phase 3	Drug dependency↓	^512^
	Vedolizumab	UC patients	Phase 1 /Phase 3	Rectal bleeding↓ Adverse reaction↓	^514,516^
	Vedolizumab	UC patients	Phase 3	Rectal bleeding↓ Frequency of defecation↓ Adverse reaction↓	^517–519^
	Vedolizumab	UC patients	Phase 2	Rectal bleeding↓ Adverse reaction↓	^520^
	Vedolizumab	UC patients	Phase 3	Rectal bleeding↓ Frequency of defecation↓ Adverse reaction↓	^521,522^
GLP-1RA	GLP-1	Male C57BL/6 mice	Animal experiment	NF-κB p65, iNOS, COX-2 ↓ ZO-1, occludin↑	^216^
SGLT-2 inhibitors	Canagliflzin	Male Wistar rats	Animal experiment	NF-κB p65, IL-6, IL-1β, MPO↓ Nrf2, PPARγ, SIRT1↑	^234^
	Empagliflozin	Male Wistar rats	Animal experiment	PI3K, AKT, TNF-α, IL-6, IL-1β↓occludin, claudin-1↑	^258^
	Dapagliflozin	Male SD rats	Animal experiment	IL-1β, IL-6, IL-18, MPO, MDA ↓ AMPK↑	^276^
NSAIDs	Diclofenac	Female C57BL/6 J mice	Animal experiment	NF-κB p65, IκB-α, iNOS↓ HO-1↑	^235^
	Aspirin	SPF male Balb/c mice	Animal experiment	JAK, STAT3, IL-6, cyclin D1↓	^311^
Mucolytic	Ambroxol hydrochloride	Male SD rats	Animal experiment	NF-κB, IL-6, IL-1β, caspase-3 ↓ IL-10↑	^236^
	Carbocisteine	Male SD rats	Animal experiment	NF-κB, IL-6, IL-1β, bax↓ bcl-2, IL-10↑	^237^
Opioid receptor agonists	Human opiorphin	Male C57BL/6 mice	Animal experiment	NF-κB, TNF-α, IL-6, IL-1β, MPO ↓ IL-10, ZO-1, claudin-1, occludin↑	^240^
PDE5 inhibitors	Sildenafil	Male SD rats	Animal experiment	NF-κB p65, COX-2, NO, MDA ↓ GSH, SOD↑	^241^
Glutamate derivatives	Thalidomide	NCM460 cells	Cell experiment	PI3K, AKT, mTOR, TNF-α, IL-6, IL-1β ↓ IL-10↑	^255^
		Male C57BL/6 mice	Animal experiment	IL-12, IFN-γ, IRF5, caspase-3 ↓ IL-10, Arg-1↑	^132^
Cholesterol absorption inhibitors	Ezetimibe	Male SD rats	Animal experiment	Akt, p-Akt, TNF-α, IL-6, CXCL10, iNOS, MDA, MPO ↓ GSH, SOD↑	^256^
S1P Antagonists	Fingolimod	Male SD rats	Animal experiment	AKT, mTOR, IL-9, MDA ↓ IL-10, TGF-β, GSH↑	^254^
BTK inhibitors	Ibrutinib	Male Albino mice	Animal experiment	PI3K, AKT, TNF-α, NF-κB, COX2↓	^257^
		Male Wistar rats	Animal experiment	L-23, IL-17, IL-6, JAK2, STAT3, MDA ↓ SOD, GSH↑	^310^
DPPIV inhibitors	Vildagliptin	Male and female Wistar rats	Animal experiment	PI3K, Akt, NF-κB, bax↓ bcl-2↑	^257^
	Linagliptin	Male Albino rats	Animal experiment	JAK2, STAT3, SOCS3, TNF-α, IL-6 ↓ IL-10↑	^309^
Acetylcholinesterase inhibitors	Donepezil	Male C57BL/6 mice	Animal experiment	NF-κB, TNF-α, IL-6, IL-1β ↓ LRP1, AMPK↑	^275^
AMPK activators	Metformin	Male Wister rats	Animal experiment	mTOR, IL-6, IL-17, IL-18 ↓ AMPK↑	^277^
		Male SD rats	Animal experiment	mTOR, IL-6, IL-1β, IL-18, TNF-α, MPO ↓ AMPK↑	^278^
α2-Adrenergic receptor agonists	Dexmedetomidine	Female C57BL/6 J mice	Animal experiment	TLR4, MyD88, p-p65, p-IκBα, TNF-α, IL-6, IL-1β↓ claudin-1, occludin, ZO-1↑	^291^
Tricyclic antidepressants	Amitriptyline	Male Wistar rats	Animal experiment	TLR4, NF-κB, IL-1β, IL-6, IL-8, IL-27, TNF-α, MPO↓	^292^
STAT3 inhibitors	Nifuroxazide	Male Wistar Albino rats	Animal experiment	IL-6, STAT3, TNF-α, MPO↓ Wnt↑	^304^
Proton Pump Inhibitors	Lansoprazole	Female SD rats	Animal experiment	MPO, MDA, NO ↓ SOD↑	^109^
Antivirals	Ganciclovir	Male C57BL/6 J mice	Animal experiment	cGAS, STING, IL-1β, IFN-γ ↓ IL-10↑	^128^
SSRI	Paroxetine	C57BL/6 J mice	Animal experiment	PGE2, GRK2, cAMP↓ EP4↑	^130^

#### Aminosalicylic acid preparations

Aminosalicylic acids are a group of nonsteroidal anti-inflammatory drugs that have analgesic and anti-inflammatory effects and can reduce inflammation and pain by suppressing the synthesis of prostaglandins. Aminosalicylic acid preparations are usually used to treat mild to moderate UC, can reduce inflammation in the colonic mucosa and may play a role in stabilizing the disease.^440^ These medications are administered orally or topically, in the form of pills, rectal suppositories, or enema fluids.^441^

Mesalazine, also known as 5-aminosalicylic acid, is an aminosalicylic acid that is usually used for UC treatment. It is mainly administered locally or by enema and is well tolerated with no obvious adverse reactions.^442^ Studies have shown that once-daily (2 g×1) or twice-daily (1 g×2) dosing is associated with 69% or 61% clinical and endoscopic remission rates, respectively. After 8 weeks of continuous dosing, the number of bowel movements was significantly reduced, the fecal calprotectin content was reduced, and the symptoms improved significantly.^443^

Sulfasalazine is a conjugate of sulfonamide and salicylic acid that may reduce the symptoms of UC by suppressing inflammation and improving immune system function.^444,445^ Its low cost and limited adverse reactions make it a commonly used drug for the treatment of UC.^446^ Studies suggest that for patients who respond poorly to mesalazine, sulfasalazine may provide an alternative that reduces inflammation and ulceration of the intestinal mucosa and possibly improves the local immune response.^447^ However, more than half of patients treated with sulfasalazine still have mild inflammation.^448^ Therefore, its combination with other inflammatory drugs could be an alternative for UC treatment.

#### Corticosteroids

Corticosteroids, also known as glucocorticoids or steroid hormones, are a class of hormones synthesized from the human adrenal cortex. They are commonly used to treat inflammatory bowel diseases such as UC.^449^ These compounds can inhibit the inflammatory response and suppress inflammation and ulceration of the intestinal mucosa, thereby relieving UC symptoms and promoting the healing of intestinal tissue. However, corticosteroids are not the preferred treatment option for long-term treatment of UC, as long-term use may cause a range of side effects, including immunosuppression, increased blood sugar, and weight gain.^450^

Budesonide, a synthetic second-generation corticosteroid drug with strong local anti-inflammatory activity, can significantly ameliorate inflammatory damage and oxidative stress^451^ and improve rectal bleeding and diarrhea.^452^ Clinically, oral budesonide (9 mg daily) is safe and effective for clinical and endoscopic remission in patients with mild to moderate UC that is refractory to mesalazine.^453^ UC patients who take budesonide twice daily show obvious improvements in clinical symptoms, a reduction in rectal bleeding, and an increase in the endoscopic remission rate.^454^

Dexamethasone is a glucocorticoid drug that is often used to reduce symptoms caused by inflammation and an overreaction of the immune system.^455^ In some cases, dexamethasone may be used to treat acute exacerbations of UC to reduce symptoms and control inflammation. Treatment with low-dose dexamethasone (10 mg/day) for 6 months allows most patients to stop taking corticosteroids and reverses drug-related adverse effects while maintaining clinical remission.^456^ Bioinformatics analysis revealed *SLC7A5* as a novel therapeutic target for the treatment of UC with dexamethasone.^457^

#### Biological agents

Biologics refers to drugs produced via modern biotechnology, usually made from substances such as proteins, peptides, or nucleic acids extracted from living organisms (e.g., bacteria, fungi, and animal cells).^458^ Biologics that are used for UC reduce intestinal inflammation by modulating the immune system, and they can inhibit specific inflammatory cytokines, such as tumor necrosis factor and other cytokines, thereby reducing the symptoms and inflammatory response in UC.^459,460^ These biologics are usually administered intravenously or subcutaneously, and their clinical application is becoming increasingly widespread in the context of the poor response of traditional drugs for the treatment of UC.^461,462^

Tofacitinib is an oral Janus kinase inhibitor approved by the U.S. Food and Drug Administration in 2018 for chronic UC treatment.^463,464^ It reduces inflammation and improves symptoms primarily by suppressing the effects of TNF-α and is typically used in patients who do not respond to or are intolerant to conventional treatments such as 5-aminosalicylic acid.^465,466^ Treatment with tofacitinib has been shown to significantly improve constipation and rectal bleeding.^467^ Among patients with moderately to severely active UC, 40.6% of patients treated with tofacitinib (10 mg twice daily) for 8 weeks achieved significant remission.^468^ In a clinical trial investigating the safety of tofacitinib, UC patients treated with tofacitinib for 16 weeks (10 mg BID) did not experience significant adverse effects.^469^ Nonetheless, for long-term treatment of UC, after the disease reaches remission, the current recommendation is that the dosage of tofacitinib should be decreased from 10 mg to 5 mg per dose.^470^

Upadacitinib is another oral Janus kinase inhibitor that plays a role in the treatment of UC by suppressing the JAK signaling pathway, thereby reducing the inflammatory response.^471,472^ It was approved by the FDA in March 2022 for moderate to severely active UC at a dose of 45 mg per day for 8 weeks and then 15 or 30 mg per day for maintenance treatment.^473^ Studies have shown that treatment with 45 mg of upadacitinib per day can quickly relieve UC symptoms, improving constipation and rectal bleeding, starting on day 1.^474^ At week 8, patients with moderate to severe UC treated with 45 mg upadacitinib showed significant improvement, with 46% reporting no intestinal urgency and 38% reporting no symptoms of abdominal pain.^475^ After 8 weeks of treatment, patients experienced a low incidence of adverse reactions as their symptoms improved.^476,477^ When UC enters the stable stage of the disease, daily treatment with 15 or 30 mg of upadacitinib has been shown to maintain the clinical remission rate with a low adverse reaction rate while greatly improving the quality of life of patients.^478–480^

Figotinib is a selective JAK1 inhibitor that regulates the activity of the immune system by inhibiting specific signaling pathways, thereby reducing inflammation and improving intestinal symptoms.^481^ In clinical trials, filgotinib has been shown to improve UC symptoms and reduce intestinal inflammation while reducing the use of hormonal drugs and alleviating the adverse effects of hormonal drugs in patients.^482^ Figotinib (200 mg or 100 mg per day) has demonstrated robust efficacy in patients with moderately to severely active UC and is well tolerated.^483^ Figotinib at a dosage of 200 mg per day is effective in treating and maintaining clinical remission in UC patients and does not exhibit corticosteroid-like dependence.^484,485^ It was demonstrated to ameliorate rectal bleeding and bowel movements within 7 days, improving the quality of life of patients.^486–488^ Notably, filgotinib has good oral availability, reaching maximum plasma concentrations at 1–3 h and plasma steady-state concentrations on day 2, making it a promising biologic agent for the treatment of UC.^489^

Mirikizumab is an antibody that functions as an IL-23p19 inhibitor and reduces the inflammatory response. It is currently in clinical trials for UC treatment.^490^ Mirikuzumab has been shown to have 48% subcutaneous bioavailability, although the bioavailability decreases with increasing BMI.^491^ Patients who received 200 mg of mirikizumab subcutaneously every 4 or 12 weeks presented a significant downward trend in the expression of genes encoding inflammatory mediators, such as cytokines and chemokines.^492^ In another 12-week study, mirikizumab demonstrated promising safety and efficacy in the treatment of UC patients, particularly in terms of endoscopic improvement, clinical remission, and histological remission, with significant therapeutic benefits.^493^ During the treatment of patients with moderate to severe UC, persistent intestinal emergencies improve and are relieved, rectal bleeding is significantly reduced,^494–496^ and C-reactive protein and fecal calprotectin levels decrease to varying degrees.^497^ Importantly, miikizumab has also shown good therapeutic results in patients who are tolerant to other drugs.^498^

Ustekinumab is an IL-12 and IL-23 antibody. It modulates the immune response by inhibiting the activity of these two cytokines, thereby reducing intestinal inflammation.^499^ Ustekinumab is an effective biologic for the treatment of ulcerative colitis, especially for patients with moderately to severely active ulcerative colitis.^500^ The study revealed that patients receiving a single intravenous dose of ustekinumab (130 mg or 6 mg/kg) followed by a maintenance dose of 90 mg subcutaneously every 8 weeks experienced a significant reduction in symptoms such as rectal bleeding and no significant adverse events.^501,502^ Clinical trials demonstrated a clinical remission rate of 18.7% at 8 weeks and 27.6% at 24 weeks in patients treated with ustekinumab, with an adverse event rate of 2.3%.^503^ Another trial confirmed that ustekinumab had a favorable safety profile when applied to UC patients for 1 year, with adverse event rates typically comparable to those of placebo.^504^ Encouragingly, the present study revealed that ustekinumab improved symptoms in UC patients as early as 7 days.^505^ After a three-year follow-up investigation, 54.1% and 56.3% of patients randomized to the ustekinumab q12w and q8w groups, respectively, achieved symptomatic relief at week 152 while on maintenance therapy.^506^ Not surprisingly, in patients with pediatric UC, ustekinumab has shown similarly favorable efficacy and safety profiles.^507^

Infliximab is an antitumor necrosis factor that reduces the inflammatory response by suppressing the action of TNF, with a bioavailability of 79.1% and a half-life of 10.8 days.^508^ Patients with severely active UC received intravenous infliximab (5 or 10 mg/kg) at weeks 0, 2, and 6, and more than 60% of patients experienced a significant response at week 8.^509^ After 8 weeks, the patient’s mucosal healing increased significantly, and symptoms such as rectal bleeding were significantly relieved.^510^ Studies have shown that the subcutaneous injection of infliximab at a dose of 120 or 240 mg every 2 weeks until the disease has stabilized for 54 weeks can achieve significant therapeutic results.^511^ In the treatment of UC in children, patients may be prone to corticosteroid dependence, and infliximab can significantly relieve this dependence.^512^ A retrospective study also confirmed that infliximab had a good therapeutic effect in patients with acute steroid-refractory UC, with surgical resection avoided in 61% of patients.^513^

Vedolizumab, a monoclonal antibody, is used primarily for the treatment of moderate-to-severe UC by targeting α4β7 integrins, blocking the homing pathway of colonic lymphocytes, and reducing the inflammatory response in the intestinal tract.^514^ This study demonstrated that vedolizumab inhibits lymphocyte homing by specifically binding to α4β7 integrins and blocking their interaction with MAdCAM-1.^515^ Among patients receiving 150 mg or 300 mg of intravenous vedolizumab, the time to clinical remission was significantly better in patients receiving 300 mg of intravenous Vedolizumab than in patients receiving 150 mg of intravenous Vedolizumab.^516^ The concentration of 300 mg intravenous vedolizumab at week 6 correlated most significantly with short- and long-term clinical remission outcomes in UC patients.^517^ At weeks 0, 2, and 6, patients treated with 300 mg of vedolizumab experienced significant clinical remission and mucosal healing, with significant reductions in symptoms such as rectal bleeding.^518,519^ In addition, in pediatric UC patients, when treated with low-dose or high-dose vedolizumab on day 1 and at weeks 2, 6, and 14, respectively, significant efficacy was similarly demonstrated.^520^ It has also been suggested that women with UC treated with vedolizumab are more likely than men to achieve early endoscopic improvement.^521^ Most importantly, vedolizumab has a favorable drug safety profile, with good bioavailability in UC patients and a low incidence of adverse events.^522,523^

Overall, biologics and monoclonal antibody-related drugs have played important roles in the treatment of ulcerative colitis, providing new therapeutic options for many patients. The development and application of biologics and targeted small-molecule drugs have significantly improved the treatment landscape for moderate-to-severe UC. JAK inhibitors (tofacitinib, upadacitinib) offer oral convenience, whereas monoclonal antibodies (influx, miikizumab) are more advantageous in terms of mucosal healing and long-term remission. Despite their effectiveness, these treatments are accompanied by potential side effects and risks. Given these characteristics and potential drawbacks, comprehensive consideration is essential. Therefore, the choice of clinical treatment needs to be weighed against a variety of factors, including efficacy, safety, and individual patient differences, to develop a better treatment plan for subsequent diagnosis and treatment.

#### Traditional Chinese medicine and its active ingredients for treating ulcerative colitis

The traditional Chinese medicines (TCMs) Pulsatilla Tang and Banxia Xiexin Tang have good therapeutic effects in relieving bloody diarrhea and abdominal pain in UC patients.^524,525^ Some commonly used TCM active ingredients include berberine and emodin, which are believed to have anti-inflammatory, antioxidant, and immunomodulatory effects that alleviate the symptoms of UC, reduce inflammation, and promote intestinal tissue repair.^526,527^ Yam polysaccharides have been shown to improve the symptoms of colitis, increase the production of IL-10, inhibit the production of inflammatory cytokines (TNF-α and IL-1β), and reduce MPO activity, thereby inhibiting intestinal inflammation and enhancing intestinal integrity.^528^ Scutellaria baicalensis polysaccharide can improve UC symptoms by regulating lipid peroxidation levels and signaling in the GPX4/ASCL4 axis, which in turn modulates cellular ferroptosis.^529^

In a UC mouse model, astragalus polysaccharides similarly regulate the levels of inflammatory factors and oxidative stress to alleviate the inflammatory response and oxidative stress in mice, maintain the integrity of the intestinal mucosal barrier, and alleviate intestinal tissue damage.^530^ In addition, dandelion can alleviate DSS-induced UC symptoms by inhibiting the MAPK signaling pathway.^531^ Moreover, dandelion polysaccharides can activate the expression of Nrf2, thereby inhibiting ferroptosis in the intestinal epithelial cells of UC model mice and restoring the integrity of the intestinal barrier.^532^ Surprisingly, the alkaloid component of aconite has also been shown to inhibit the aberrant expression of inflammatory cytokines (TNF-α, IL-1β, IL-6, and IFN-γ) through the MAPK/NF-κB/STAT3 signaling pathway, alleviating the symptoms of UC.^533^

#### Fecal microbiota transplantation

Fecal microbial transplantation (FMT), also known as fecal microbiota transplantation, is an emerging treatment for gut microbial dysbiosis.^534^ In the course of clinical trials, FMT significantly improved the levels of fecal calprotectin and C-reactive protein in UC patients and played a role in the adjuvant treatment of UC.^535^ After FMT treatment, the proportion of proinflammatory Enterobacteriaceae decreased, the abundance of Collinsella increased, and the expression of IL-6 and IL-1β significantly decreased; the efficacy of FMT was found to last for more than 1 year.^536^ Notably, this study revealed that FMT under hypoxic and frozen conditions had better effects than did FMT under conventional conditions.^537,538^ The cost of FMT for the treatment of active UC is relatively low, and the benefits are substantial, but its safety needs to be verified by further clinical trials.^539–541^

## Conclusions and prospects

The prevalence of UC has been increasing each year, and symptoms such as perennial bloody diarrhea in UC patients have severely affected the quality of life of patients, resulting in UC becoming a serious and pervasive global public health problem. Therefore, many researchers and medical scientists have opted to conduct UC research. On the basis of the expanding volume of currently emerging research, we have summarized several relevant targets and signaling pathways of UC, including the PI3K/AKT, NF-κB, and Wnt/β-catenin pathways, as well as the contributions of lncRNAs and miRNAs, endoplasmic reticulum stress, mitochondrial dysfunction, mesenchymal stem cells, macrophage polarization, the gut microbiota, and many other targets and pathways (Table 2).

**Table 2:** Key targets and signaling pathways involved in UC

Signaling pathways and targets	Mechanisms of action	Subjects	Target proteins	References
Autophagy	Inhibits inflammatory factors, protects intestinal barrier	Male C57BL/6 mice	circ HECTD1/miR-182-5p	^85^
		Esrra ^+^ /^+^ and esrra ^−^ /^−^ mice	AMPK	^83^
		Male C57BL/6 mice	AMPK	^84^
		Male SD rats	LC3, P62	^88,89^
	Protects intestinal barrier	Male BALB/c mice	JNK	^87^
ER stress	Protects intestinal barrier	Mendelian randomization	GPX1	^92^
	Inhibits inflammatory factors, protects intestinal barrier	UC patients	PERK, IRE1	^95^
	Protects intestinal barrier	UC patients	IRE-1	^96^
	Inhibits inflammatory factors, protects intestinal barrier	UC patients	PAR2, PAR4	^97^
		Female Pcyt1aLoxP/LoxP mice	Muc2	^98^
		C57BL/6 mice	Muc2	^99^
		Male C57BL/6 mice	ERK1/2	^100^
		SPF C57BL/6 mice	omentin-1	^101^
Mitochondria pathway	Inhibits inflammatory factors	WT male C57BL/6 J mice	PARP1	^103^
	Protects intestinal barrier	Male C57BL/6 J mice	PER2	^104^
		Male C57BL/6 mice	ACAT1	^105^
	Inhibits inflammatory factors, protects intestinal barrier	HSF2 ^−^ /^−^ mice	PARL/PINK1/Parkin axis, mtROS	^106^
Oxidative stress	Inhibits inflammatory factors, protects intestinal barrier	Male C57BL/6 mice	GSH, SOD, CAT	^107^
		Male C57BL/6 mice	GSH	^108^
		Female SD rats	GSH, SOD, CAT	^109^
Macrophage polarization	Inhibits inflammatory factors, protects intestinal barrier	Male C57BL/6 mice	TFF2	^114^
	Inhibits inflammatory factors	Male C57BL/6 mice	S1PR1	^116^
		WT C57BL/6 J mice	M2	^117^
	Inhibits inflammatory factors, regulating gut microbiota	WT male C57BL/6 mice	*C. butyricum*	^118^
	Inhibits inflammatory factors, protects intestinal barrier	Male C57BL/6 mice	miR-21a-5p	^119^
	Protects intestinal barrier	C57BL/6 mice	miR-590-3p, YAP/ β-catenin	^120^
	Inhibits inflammatory factors, protects intestinal barrier	Human Colonic Epithelial Cells	IRF4	^121^
	Inhibits inflammatory factors	BALB/c mice and Male C57BL/6 mice	M1, M2	^122–124^
	Inhibits inflammatory factors, protects intestinal barrier	BALB/c mice	CD206, Arg1	^125^
	Inhibits inflammatory factors, regulating gut microbiota	Male C57BL/6 mice	Lactic acid synthesis	^127^
	Inhibits inflammatory factors, protects intestinal barrier	Male C57BL/6 J mice	cGAS-STING	^128^
		Male C57BL/6 mice	IRF5	^129^
	Inhibits inflammatory factors	C57BL/6 J mice	GRK2/cAMP/CREB axis	^130^
Mesenchymal stem cells	Inhibits inflammatory factors, protects intestinal barrier	Male C57BL/6 mice	EMSCs	^132^
		C57BL/6 mice	Nrf2/Keap1/ARE axis	^134^
		SPF male SD rats	PD-L1	^135^
	Protects intestinal barrier	SPF male SD rats	HIF-1α	^136^
	Inhibits inflammatory factors	Male BALB/c rats	IL-6	^137^
	Inhibits inflammatory factors	Male SD rats	HGF	^138^
	Inhibits inflammatory factors, protects intestinal barrier	Male C57BL/6 mice	ADMSCs	^139^
NF-κB	Inhibits inflammatory factors, protects intestinal barrier, regulates intestinal microbes	Male C57BL/6 mice	AKT	^213^
	Inhibits inflammatory factors, protects intestinal barrier	SPF C57BL/6 J mice	NF-κBp65	^215^
		Wistar male rats	TLR4/Myd88	^216^
	Inhibits inflammatory factors	Female C57BL/6 mice	IκBα	^217^
	Inhibits inflammatory factors	Female WT mice	RIPK1	^218^
		Male C57BL/6 mice	IL-6	^219^
	Inhibits inflammatory factors, protects intestinal barrier	Male C57BL/6 J mice	TRIM8	^220^
	Inhibits inflammatory factors, protects intestinal barrier, regulates intestinal microbes	Male C57BL/6 J mice	NF-κB p65	^221^
	Protects intestinal barrier	Male C57BL/6 J mice	NF-κB p65	^222,223^
	Inhibits inflammatory factors	Male SD rats	TFF-3, GLP-1	^224^
		Male Wistar rats	SIRT1/Nrf2 axis	^225^
		Male Wistar rats	TLR4	^226^
		Female SD rats	p-p65	^227^
	Inhibits inflammatory factors, protects intestinal barrier	Female BALB/c mice	IκBα	^228^
		Male Kunming mice	NF-κB p65	^229^
		C57BL/6 J male mice	NF-κB p65	^230^
	Protects intestinal barrier	SD rats	NF-κB p65 and p52	^231^
	Inhibits inflammatory factors, protects intestinal barrier	Male C57BL/6 J mice	IκBα, p65	^232^
	Inhibits inflammatory factors	Male C57BL/6 mice	IκBα	^233^
		Male Wistar rats	TLR4/MAPK	^234^
	Inhibits inflammatory factors, protects intestinal barrier	Female C57BL/6 J mice	NF-κB p65, IκB-α	^235^
		Male SD rats	IκB-α	^236,237^
		Male Wister rats	TLR4	^238^
	Inhibits inflammatory factors	Male Wister rats	TLR4	^239^
	Inhibits inflammatory factors, protects intestinal barrier	Male C57BL/6 mice	NF-κB p65	^240^
	Inhibits inflammatory factors	Male SD rats	NF-κB p65	^241^
PI3K/Akt	Protects intestinal barrier	C57BL/6 mice	AKR1B10	^249^
	Inhibits inflammatory factors	C57BL/6 mice	oxytocin	^250^
		PBMCs	PI3K	^252^
	Inhibits inflammatory factors, protects intestinal barrier	Male C57/BL6 mice	Akt	^251^
	Protects intestinal barrier	Male C57B6 mice	TGR5	^253^
	Inhibits inflammatory factors	NCM460 cells	PI3K/Akt	^255^
		Male SD rats	Akt	^256^
		Male SD rats	Akt	^254^
	Inhibits inflammatory factors, protects intestinal barrier	Male and female Wistar rats	PI3K/Akt	^257^
	Protects intestinal barrier	Male Wistar rats	SIRT1	^258^
	Inhibits inflammatory factors	Male ICR mice	PI3K/Akt	^259^
Wnt	Protects intestinal barrier	C57BL/6 mice	Wnt	^263^
		Male C57B6 mice	Wnt-5a	^264^
		Male C57BL/6 J mice	Wnt/β-catenin	^265^
	Inhibits inflammatory factors, protects intestinal barrier	Male BALB/c mice	miR-182-5p	^266^
	Protects intestinal barrier	Male C57/BL mice	GABAAR	^267^
	Delaying disease progression	Male WT C57BL/6 mice	Cldn-7	^268^
		Male C57BL/6 mice	CYP24A1	^269^
AMPK	Inhibits inflammatory factors	Male C57BL/6 mice	p-AMPK	^272^
	Protects intestinal barrier	WT C57BL/6 mice	P2Y1R	^274^
	Inhibits inflammatory factors, protects intestinal barrier	Male C57BL/6 mice	LRP1	^275^
	Inhibits inflammatory factors	Male SD rats	NF-κB	^276^
		Male Wister rats	p-AMPK	^277^
		Male SD rats	p-AMPK	^278^
TLRs	Inhibits inflammatory factors, protects intestinal barrier	C57BL/6 J mice	TLR	^282^
		Female BALB/c mice	TLR4	^283^
	Regulating gut microbiota	WT C57BL/10 J mice	TLR4	^284^
	Inhibits inflammatory factors, protects intestinal barrier	Male C57BL/6 J mice	NLRP6	^285^
		Male BALB/c mice	RIP3	^286^
		Male Wistar rats	P2X7R	^287^
		Female C57BL/6 J mice	TLR4/Myd88	^291^
	Inhibits inflammatory factors	Male Wistar rats	TLR4	^292^
		SPF male C57BL/6 J mice	TLR4	^293^
	Inhibits inflammatory factors, protects intestinal barrier	Male C57BL/6 mice	IRAK1/4	^294^
	Inhibits inflammatory factors	WT C57BL/6 J mice	MTDH	^295^
	Inhibits inflammatory factors, protects intestinal barrier	C57BI/6 mice	TLR4	^296^
STATs	Inhibits inflammatory factors	C57BL/6 mice	STAT3	^303^
	Inhibits inflammatory factors, protects intestinal barrier	Female BALB/c mice	SPHK-1/S1PR1/STAT3 axis	^305^
		Male C57BL/6 mice	IL-22/STAT3	^306^
		Male albino rats	IL-6/STAT3	^307^
		Male Wistar Albino rats	IL-6/STAT3	^304^
		Male C57BL/6 J mice	JAK2/STAT3	^308^
		Male SD rats	STAT3	^309^
	Inhibits inflammatory factors	Male Wistar rats	JAK2/STAT3	^310^
		SPF male Balb/c mice	IL-6/JAK/STAT3 axis	^311^
		Male albino rats	JAK2/STAT3	^312^
		Male C57BL/6 mice	IL-4/STAT6	^313^
	Inhibits inflammatory factors, protects intestinal barrier	Male BALB/c mice	STAT6	^314^
LncRNAs	Protects intestinal barrier	Male C57BL/6 mice	LncRNA TUG1	^319^
	Protects intestinal barrier	Fetal human cells	LncRNA SNHG5	^320^
	Delaying disease progression	Human Colonic Epithelial Cells	LncRNA PMS2L2	^321^
		C57BL/6 J mice	LncRNA PINT	^322^
	Protects intestinal barrier	Caco-2 cells	LncRNA GATA6-AS1	^323^
	Protects intestinal barrier	Intestinal epithelial cells	LncRNA NEAT1	^324^
	Inhibits inflammatory factors, protects intestinal barrier	Female BALB/c mice	LncRNA MIR4435-2HG	^325^
		Fetal human cells	LncRNA 78583	^328^
	Inhibits inflammatory factors, protects intestinal barrier	SD rats	LncRNA MEG3	^326^
		Male C57BL/6 mice	LncRNA MEG3	^327^
		Fetal human cells	LncRNA UCA1	^318^
miRNAs	Inhibits inflammatory factors	Biopsy specimens	miR-21, miR-433, miR-590	^333^
		C57/BL6 mice	miR-124	^334^
	Inhibits drug resistance	Male SD rats	miR-145	^337^
	Protects intestinal barrier, delaying disease progression	WT C56BL/6NJ mice	miR-24-3p	^340^
		BALB/c mice	miR-199a-3p	^341^
		Male SD rats	miR-29a-3p	^342^
	Inhibits inflammatory factors, protects intestinal barrier	SD rats	miR-939, miR-376a	^336^
		Male C57BL/6 mice and WT mice	miR-195-5p	^343,344^
	Inhibits inflammatory factors, protects intestinal barrier	Male Wistar rats	miR-200b-3p	^335^
		Male SD rats	miR-330	^345^
	Inhibits inflammatory factors	Female C57BL/6 mice	miR-29c-3p	^346^
	Protects intestinal barrier	C57BL/6 mice	miR-182-5p	^347^
	Inhibits inflammatory factors, protects intestinal barrier	C57BL/6 mice	miR-181a	^348^
		Male C57BL/6 J mice	miR574-5p	^349^
	Inhibits inflammatory factors, delaying disease progression	C57BL/6 and BALB/c nude mice	miR149-3p	^350^

Recently, new targets and signaling pathways that target UC, including ferroptosis, inflammasomes, and the Th/Treg cell balance, have also been shown to have good efficacy in UC treatment and have good potential for development. Although research on these new targets has not yet entered the phase of clinical trials, they will most likely become the mainstay of treatment for UC in the future (Table 3). Unfortunately, despite the remarkable progress in preclinical studies, research on these targets and signaling pathways has mainly remained in the animal and cellular experiments stage and has not yet entered the clinical trials stage. Translating these research results into clinical practice still requires overcoming multiple challenges, such as individualized treatment, long-term safety and patient compliance, which is a long process.

**Table 3:** Novel signaling pathways and pharmacological targets involved in UC

Signaling pathways and targets	Type of research	Subjects	Target proteins	References
Ferroptosis	Bioinformatics analysis	——	CD44, MUC1	^354^
	Animal experiment	Female C57BL/6 mice	LCN2	^353^
		SPF male C57BL/6 mice	Gpx4	^355^
		Male C57BL/6 mice	Nrf2/Gpx4	^356^
		Male C57BL/6 mice	AMPK	^358^
		Male C57BL/6 WT mice	MELK	^360^
		SPF C57BL/6 mice	ACSF2	^361^
	Metabolomics	Biopsy specimens	Nrf2, Gpx4, CYP1A1	^357^
	Animal experiment	Male C57BL/6 J mice	Gpx4	^362^
Inflammasome	Animal experiment	SPF male C57BL/6 mice	NLRP3	^365^
		SPF male SD rats	NLRP3	^366^
	Animal experiment	Male SD rats	Nrf2/NLRP3	^367^
		Male SD rats	NLRP3	^368^
		Female C57BL/B6 mice	GPR84/NLRP3	^369^
		C57BL/6 mice	RNF31/NLRP3	^370^
	Cellular experiment	SW480 cells	DLG2/NLRP3	^371^
	Animal experiment	Male C57BL/6 mice	HSF2/NLRP3	^372^
		Female BalB/c mice	NLRP3	^373^
		SD rats	TXNIP/NLRP3	^374,375^
		Male Wistar rats	NLRP3	^376^
		SPF male C57BL/6 J mice	FXR-NLRP3	^377^
		Male C57BL/6 J mice	NF-κB/NLRP3	^378^
		C57BL/6 J mice	NLRP3	^379^
		C57BL/6 mice	Mist1/NLRP3	^380^
		Male BALB/c mice	NFATC1/NLRP3	^381^
		Male ICR mice	Nrf2/NLRP3	^382^
Th/Treg cell balance	Animal experiment	SPF male Balb/c mice	JAK1/STAT3/SOCS axis	^386^
		Female C57BL/6 mice	CK2/SIRT1	^387^
		Male SD rats	PD-L1	^388^
		SD rats	ST6GAL1	^389^
		SPF male Balb/c mice	JMJD3	^390^
		C57BL/6 mice	*Lactobacillus*	^391^
		Female C57BL/6 mice	PDE9A	^392^

We have also summarized the drugs that are clinically used for UC treatment, including mainly aminosalicylic acid preparations, corticosteroids and biologics. Unfortunately, most biologics are still in clinical trials, although further efforts are underway to develop safe and durable treatment strategies. Moreover, lifestyle interventions, such as reasonable diets and timely nutritional supplementation, should be strengthened. Considering the complexity of the pathogenesis of UC and the characteristics of UC prolongation and refractory healing, providing patients with a personalized treatment plan is necessary.

In the future, with the in-depth study of UC disease mechanisms and the development of more innovative drugs, more precise and personalized treatments with multiple targets, such as the combination of conventional drugs, conventional drugs and biologics, and biologics and biologics, are expected to be developed. At the level of the regulatory mechanism of drug targets, the anti-TNF-α antibody adalimumab significantly inhibits the NF-κB and MAPK signaling cascade response by blocking the binding of TNF-α to the receptor,^542^ whereas the ustekinumab antibody, which targets the IL-12/IL-23 axis, accurately inhibits the differentiation of Th1/Th17 cells and reduces the levels of IL-17 and IFN-γ by targeting its shared p40 subunit.^543^ The JAK-STAT pathway serves as a hub for cytokine signaling, and inhibitors such as tofacitinib effectively block IL-6- and IL-23-mediated immune cell activation by competitively occupying the ATP binding site of the kinase.^544^ In addition, emerging targets such as IL-36 pathway inhibitors promote Treg cell proliferation by neutralizing the proinflammatory effects of IL-36, and TLR4/MyD88 small molecule inhibitors provide a new way of treating UC by modulating innate immune hyperactivation triggered by colony dysregulation.^545^ In the development of drugs for UC, in addition to continuing to optimize existing drug targets and therapeutic strategies, we should also focus on the development of drugs with novel mechanisms of action. For example, drug development targeting the repair of intestinal barrier function can promote the proliferation and differentiation of intestinal epithelial cells and increase the expression of intercellular tight junction proteins to fundamentally improve intestinal barrier function and reduce the occurrence of inflammation. Alternatively, it is possible to modulate the host immune response and alleviate UC inflammation by altering the composition of the intestinal microbiota, such as through supplementation with specific probiotics or the development of analogs of microbial metabolites.

To improve the safety and effectiveness of UC treatment, future research needs to better understand the pathogenic mechanisms related to the inflammatory response and intestinal epithelial barrier damage in the treatment and progression of UC disease. A more comprehensive understanding of the pathways and processes of UC will facilitate the development of new therapeutic targets and improve existing treatment methods, with the ultimate goal of improving the quality of life of UC patients.
